# Human lncRNA *SUGCT-AS1* Regulates the Proinflammatory Response of Macrophage

**DOI:** 10.3390/ijms241713315

**Published:** 2023-08-28

**Authors:** Yeong-Hwan Lim, Gwangho Yoon, Yeongseo Ryu, Dahee Jeong, Juhyun Song, Yong Sook Kim, Youngkeun Ahn, Hyun Kook, Young-Kook Kim

**Affiliations:** 1 Basic Research Laboratory for Vascular Remodeling, Chonnam National University Medical School, Hwasun 58128, Republic of Korea; 2 Department of Biochemistry, Chonnam National University Medical School, Hwasun 58128, Republic of Korea; 3 BioMedical Sciences Graduate Program (BMSGP), Chonnam National University, Hwasun 58128, Republic of Korea; 4 Division of Brain Disease Research, Department for Chronic Disease Convergence Research, Korea National Institute of Health, Cheongju 28159, Republic of Korea; 5 Department of Anatomy, Chonnam National University Medical School, Hwasun 58128, Republic of Korea; 6 Cell Regeneration Research Center, Chonnam National University Hospital, Gwangju 61469, Republic of Korea; 7 Biomedical Research Institute, Chonnam National University Hospital, Gwangju 61469, Republic of Korea; 8 Department of Cardiology, Chonnam National University Medical School, Gwangju 61469, Republic of Korea; 9 Department of Pharmacology, Chonnam National University Medical School, Hwasun 58128, Republic of Korea

**Keywords:** long non-coding RNAs, *SUGCT-AS1*, hnRNPU, *MALT1*, macrophage, vascular smooth muscle cells, inflammation

## Abstract

Macrophages are the major primary immune cells that mediate the inflammatory response. In this process, long non-coding RNAs (lncRNAs) play an important, yet largely unknown role. Therefore, utilizing several publicly available RNA sequencing datasets, we predicted and selected lncRNAs that are differentially expressed in M1 or M2 macrophages and involved in the inflammatory response. We identified *SUGCT-AS1*, which is a human macrophage-specific lncRNA whose expression is increased upon M1 macrophage stimulation. Conditioned media of *SUGCT-AS1*-depleted M1 macrophages induced an inflammatory phenotype of vascular smooth muscle cells, which included increased expression of inflammatory genes (*IL1B* and *IL6*), decreased contractile marker proteins (ACTA2 and SM22α), and increased cell migration. Depletion of *SUGCT-AS1* promoted the expression and secretion of proinflammatory cytokines, such as *TNF*, *IL1B*, and *IL6*, in M1 macrophages, and transcriptomic analysis showed that *SUGCT-AS1* has functions related to inflammatory responses and cytokines. Furthermore, we found that *SUGCT-AS1* directly binds to hnRNPU and regulates its nuclear–cytoplasmic translocation. This translocation of hnRNPU altered the proportion of the *MALT1* isoforms by regulating the alternative splicing of *MALT1*, a mediator of NF-κB signaling. Overall, our findings suggest that lncRNAs can be used for future studies on macrophage regulation. Moreover, they establish the *SUGCT-AS1*/hnRNPU/*MALT1* axis, which is a novel inflammatory regulatory mechanism in macrophages.

## 1. Introduction

Cardiovascular diseases are accompanied by various dysfunctions, including hyperactivation of vascular endothelial cells, infiltration and inflammation of macrophages, and dedifferentiation of vascular smooth muscle cells (VSMCs) into an abnormal inflammatory phenotype [1,2,3,4]. Notably, monocytes infiltrated from the blood into the vascular wall by endothelial cell activation differentiate into macrophages and polarize to either classically or alternatively activated macrophages, also called M1 or M2 macrophages, respectively [4,5]. In addition, these macrophages form foam cells together with VSMCs following stimuli, including cholesterol and LDL, which thicken the inner wall of the blood vessels and lead to plaque development [2,6]. Thus, macrophages play critical roles during this chronic inflammatory progression.

Polarized macrophages can stimulate VSMCs by secreting various soluble signaling molecules, including cytokines and chemokines [7,8]. Hence, it is necessary to derive results through interaction studies between various vascular cell types in studying inflammatory response. Many researchers have made significant contributions to this research area by identifying numerous related mechanisms using animal models (ApoE^−/−^ mice and LDLr^−/−^ mice) of atherosclerosis, which have been induced by the knockout of specific genes, including Apolipoprotein E (ApoE) and LDL-receptor (LDLr) [9,10,11]. However, there are some differences between animal disease models and human disease mechanisms, which limits the progress of human research on inflammation and atherosclerosis [12]. To overcome these limitations, it is also necessary to approach mechanism studies utilizing various publicly available human datasets related to inflammation, atherosclerosis, and vascular cells.

Non-coding RNAs (ncRNAs) are a group of RNA transcripts, which rarely encode proteins, and representative regulatory ncRNAs, including microRNAs (miRNAs), long non-coding RNAs (lncRNAs), and circular RNAs (circRNAs) [13]. They occupy the genome more than protein-coding RNAs in humans and rodents [14]. Moreover, ncRNAs are providing new insights into many diseases and biological processes, which have not been elucidated through protein-coding genes [13,15]. Unlike miRNAs, the functions of many lncRNAs and circRNAs in inflammatory- or atherosclerosis-related responses in macrophages are less well characterized [16]. Indeed, lncRNAs are a class of non-coding RNAs longer than 200 nucleotides that can control the expression of protein-coding genes through various mechanisms [15]. One of the well-characterized lncRNAs, *MAARS* (macrophage-associated atherosclerosis lncRNA sequence), is macrophage-specific and binds to the RNA-binding protein—HuR—to regulate apoptosis and efferocytosis of macrophages in atherosclerotic lesions, independent of lipid accumulation and inflammatory signaling pathways [17]. Another lncRNA, *MacORIS* (macrophage-enriched obesity-associated long intergenic non-coding RNA serving as a repressor of IFN-γ signaling), is human macrophage-specific and regulates the interferon γ (IFN-γ) signaling-induced Janus kinase 2 (JAK2) alongside the phosphorylation of signal transducer and activator of transcription 1 (STAT1) in THP-1 macrophages [18]. Although several lncRNAs have been shown to be involved in the biological functions of macrophage and atherosclerotic processes, studies of lncRNAs in these processes are still insufficient.

In this study, we analyzed various public RNA sequencing data on human atherosclerosis and macrophages to screen differentially expressed lncRNAs in these samples. We also confirmed and characterized that the lncRNA *SUGCT-AS1*/hnRNPU complex could modulate the inflammatory response in macrophages through the regulation of alternative splicing of *MALT1*, a key mediator in the NF-κB signaling pathway. We found the phenotypic effect of cytokines secreted from these *SUGCT-AS1*-depleted macrophages on VSMC inflammation, which indicates a novel regulatory mechanism between macrophages and VSMCs through lncRNA regulation.

## 2. Results

### 2.1. Identification of lncRNAs Involved in the Macrophage Polarization and the Progression of Atherosclerosis

**Figure 1:**
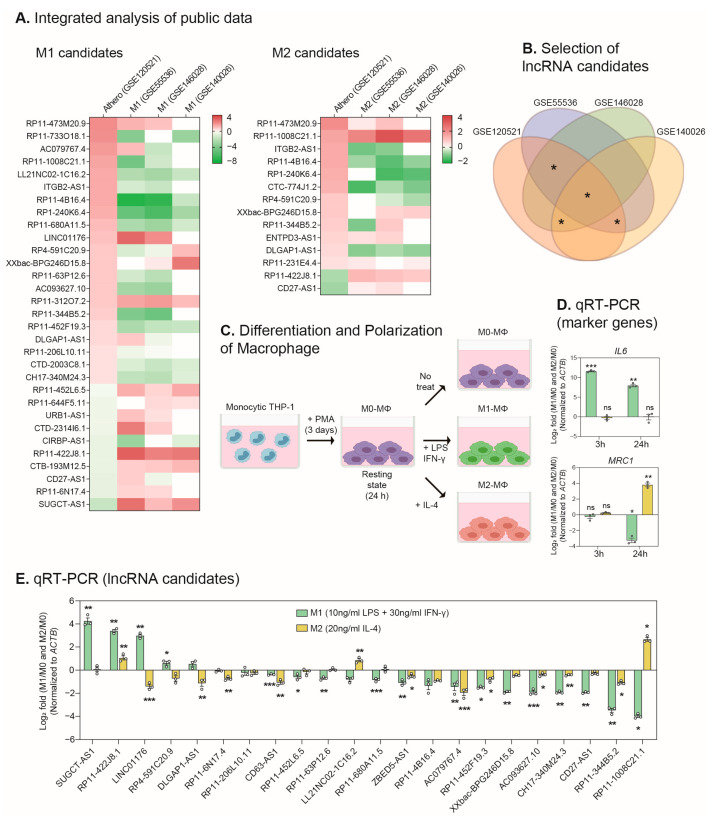
Identification of lncRNA candidates through integrative transcriptome analysis and experimental validation. (**A**) Comprehensive analysis of expression profiles of lncRNA candidates during atherosclerosis progression and macrophage activation (M1 and M2 macrophages). The criteria for the selection of differentially expressed lncRNAs are described in the Section 4. (**B**) The combination of atherosclerosis data and three macrophage datasets is shown as a Venn diagram. Candidates for lncRNAs that showed significant expression patterns at the intersections (asterisks) between the datasets were selected. (**C**) The processes of differentiation from human monocytic THP-1 cells into M0 macrophages and polarization into M1 or M2 macrophages. Monocytic THP-1 cells were cultured with PMA (100 nM) for three days. After resting for one day, M1 (10 ng/mL LPS and 30 ng/mL IFN-γ) or M2 (20 ng/mL IL-4) stimulation was administered. (**D**) qRT-PCR measurement of the expression of pro- and anti-inflammatory genes (*IL6* and *MRC1*) in M1 and M2 macrophages activated for 3 and 24 h (*n* = 3). (**E**) qRT-PCR measurement of the expression of lncRNA candidates in M1 and M2 macrophages activated for 24 h (*n* = 3). The expression of inflammatory genes and lncRNAs was normalized to *ACTB*. The circles in the bars indicate each data point. Data are presented as mean ± SEM. An unpaired two-tailed *t*-test with Welch’s correction was used for statistical analysis. * *p* < 0.05, ** *p* < 0.01, *** *p* < 0.005, ns: not significant.

To screen lncRNA candidates related to atherosclerosis and macrophage polarization, we interrogated human lncRNAs collated from the atherosclerosis progression dataset (GSE120521) and the three datasets on the M1 and M2 macrophage, activated from the human CD14^+^ monocyte-derived macrophage (GSE55536, GSE146028, GSE140026) in the GEO database (Figure 1A, see Section 4) [19,20,21,22]. For the atherosclerotic dataset (GSE120521), changes in expression were measured in stable plaques from patients with early atherosclerosis versus unstable plaques with worsening lesions. First, we screened for lncRNAs that were significantly increased or decreased in stable plaques versus unstable plaques. For each of these filtered lncRNAs, we selected only those lncRNAs that were significantly changed in at least two of the three datasets profiled in the samples of activated macrophages (GSE55536, GSE146028, GSE140026) (the groups of selected lncRNAs are indicated in Figure 1B). Thus, 31 and 14 lncRNA candidates were selected from the M1 and M2 macrophages, respectively, and 11 of them showed changes in both the M1 and M2 (Figure 1A and Figure S1A). Among these candidates, lncRNAs that have been studied previously, such as *MIR155HG*, *Lnc-DC*, and *RP11-184M15.1*, were similarly identified from our analysis (Figure S1B) [18,23,24,25,26]. This suggests that our integrated analysis is an appropriate approach for discovering lncRNAs related to atherosclerosis and macrophages. To verify the expression patterns of the identified lncRNAs, we used macrophages (M0) differentiated from the THP-1 human monocytic cell line. We also activated the M0 macrophages into either the M1 or M2 type using LPS and IFN-γ or IL-4, respectively (Figure 1C). We confirmed that *IL6*, a proinflammatory marker, was significantly increased following LPS and IFN-γ treatment for 3 or 24 h. Conversely, the anti-inflammatory marker, *MRC1*, was decreased when the cells were treated with LPS and IFN-γ; however, it increased when treated with IL-4 for 24 h (Figure 1D). When we measured the expression changes of 22 lncRNA candidates, most candidates showed a significant change in their expression from these macrophage activation models (Figure 1E).

### 2.2. SUGCT-AS1 Is a Human Macrophage-Enriched Nuclear lncRNA

**Figure 2:**
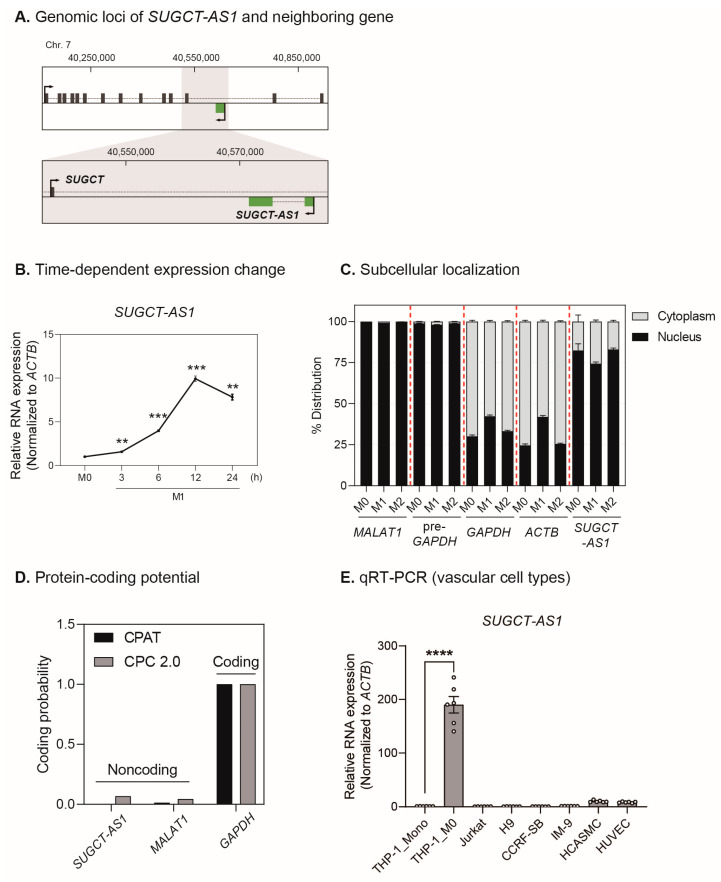
Characterization of *SUGCT-AS1* involved in the differentiation and polarization of macrophages. (**A**) Genomic loci of lncRNA *SUGCT-AS1* and its neighboring protein-coding gene *SUGCT* in the human genome (GRCh37/hg19). The genomic information was obtained from the UCSC Genome Browser. (**B**) qRT-PCR measurement of *SUGCT-AS1* in THP-1-derived macrophages administered with M1 stimuli (10 ng/mL LPS and 30 ng/mL IFN-γ) at different treatment times (*n* = 3). (**C**) Subcellular localization of *SUGCT-AS1* in THP-1-derived macrophages of each subtype (*n* = 3). *MALAT1* and pre-*GAPDH* were used as controls for nuclear RNA, and *ACTB* and *GAPDH* were used as controls for cytoplasmic RNA. (**D**) The protein-coding potential of *SUGCT-AS1*. The coding probability of *SUGCT-AS1* was assessed by CPAT and CPC 2.0 tools. *MALAT1* was used as a control for the non-coding RNA, and *GAPDH* was used as a control for the protein-coding RNA. (**E**) qRT-PCR measurement of vascular cell type-specific expression of *SUGCT-AS1* (*n* = 6). The circles in the bars indicate each data point. THP-1_mono: monocytic THP-1 cells, THP-1_M0: THP-1-derived macrophages, Jurkat and H9: T-cell lines, IM-9 and CCRF-SB: B-cell lines, HCASMCs: human coronary artery smooth muscle cells, HUVECs: human umbilical vein endothelial cells. The RNA expression was normalized to *ACTB* (**B**,**E**). Data are presented as mean ± SEM. An unpaired two-tailed *t*-test with Welch’s correction was used for statistical analysis. ** *p* < 0.01, *** *p* < 0.005, **** *p* < 0.001.

Among the 22 lncRNA candidates identified above, we selected *SUGCT* antisense transcript 1 (*SUGCT-AS1*, also known as *AC004988.1*; ENST00000415237) for further experiments (Figure 1E). *SUGCT-AS1* was most highly upregulated in M1 macrophages, suggesting that *SUGCT-AS1* may have an M1-specific functional role. There are no previous functional studies of *SUGCT-AS1*, other than the study where it promoted ovarian cancer metastasis through its role as a sponge for miR-101 [27]. *SUGCT-AS1* (chr7:40,577,726–40,586,527) is a human-specific lncRNA that is transcribed in the antisense direction on the intron of the succinyl-CoA–glutarate-CoA transferase (*SUGCT*), and it is not conserved in other species, such as mice (Figure 2A). The analysis of the sequencing reads obtained from the RNA-seq of THP-1-derived macrophages showed that the 5′ and 3′ ends of the *SUGCT-AS1* transcript annotated in the genome database matched the RNA-seq signals (Figure S2). In addition, PCR amplification with the primers designed at both ends of these RNA-seq signals confirmed that only one isoform identical to the transcript present in the database exists near the *SUGCT-AS1* locus (Figure S3). The reverse transcription with oligo-d(T) or random hexamer primers, respectively, suggests that *SUGCT-AS1* might have a poly(A) tail structure (Figure S4). To confirm whether *SUGCT-AS1* also responds to other inflammatory stimuli, we administered TNFα, IL-6, and oxidized low-density lipoprotein (oxLDL) to the cells (Figure S5A,B). Unlike *TNF* and *CXCL10*, *SUGCT-AS1* responded only to LPS and IFN-γ (Figure S5A). In addition, the expression of oxLDL-related genes was changed by oxLDL, which induced the macrophage into foam cells, but *SUGCT-AS1* did not respond (Figure S5B). This suggests that *SUGCT-AS1* is not directly affected by oxLDL, a key factor in inducing atherosclerosis, but is involved in the function of inflammatory M1 macrophages. Next, the cytokines for M1 or M2 stimulation were treated for 0 to 24 h to investigate the time-dependent expression changes, which were verified by analyzing the expression of each macrophage subtype marker. The M1 markers *TNF*, *IL1B*, and *IL6* were strongly expressed at relatively early stimulation times (3–6 h, Figure S6A). Conversely, the M2 markers *IL10*, *MRC1*, and *CD200R1* were strongly expressed at the late stimulation time (24 h, Figure S6A). In a time-dependent manner, *SUGCT-AS1* expression was significantly increased with M1 stimulation (Figure 2B). Cellular fractionation experiments in all subtypes of macrophages revealed that *SUGCT-AS1* was predominantly localized within the nucleus (Figure 2C). Bioinformatics tools were used to evaluate whether *SUGCT-AS1* encodes a protein, and the results confirmed that *SUGCT-AS1* is a non-coding RNA in both tools (Figure 2D). Finally, to determine the potential relevance of *SUGCT-AS1* for inflammatory response, its cell specificity was evaluated. Evaluation in relevant cell types, including THP-1 monocytic cells, M0 macrophages, T-cells, B-cells, HCASMCs, and HUVECs, demonstrated that *SUGCT-AS1* expression is very high in M0 macrophages but slightly expressed in other vascular cell types (Figure 2E). In addition, markers (*CD14*, *CD3E*, *CD19*, *TAGLN*, and *vWF*) for each type were used to confirm the cell specificity of each cell type (Figure S7). Overall, the human nuclear lncRNA *SUGCT-AS1* is enriched in macrophages and its expression is enhanced by M1 stimulation, suggesting that *SUGCT-AS1* may have an inflammation-related function.

We further investigated whether additional lncRNA candidates were also changed in macrophage activation. Expressions of *LINC01176* and *RP11-1008C21.1* were increased only by each of the M1 and M2 stimuli, respectively, while *DLGAP1-AS1* and *LL21NC02-1C16.2* showed decreased responses to all stimuli (Figure S6B). Furthermore, *RP4-591C20.9* and *RP11-422J8.1* were increased by M1 stimulation and decreased by M2 stimulation. Cellular fractionation experiments on all subtypes of the macrophages revealed that four lncRNA candidates (*LL21NC02-1C16.2*, *LINC01176*, *RP4-591C20.9*, *DLGAP1-AS1*) were predominantly localized within the nucleus, while *RP11-422J8.1* resided in the cytoplasm (Figure S8A). *RP11-1008C21.1* was identified only in M2 macrophages and is relatively evenly distributed within the nucleus and cytoplasm. Through evaluation of vascular cell specificity, it was confirmed that the expression of all six lncRNA candidates was significantly enhanced in THP-1-derived M0 macrophages (Figure S8B). *DLGAP1-AS1* was revealed to be more strongly expressed in HCASMCs and HUVECs than in monocytes and macrophages, suggesting that it may have a main function in both macrophages and other vascular cells. Finally, it was also confirmed by bioinformatics tools that these six lncRNA candidates do not encode proteins (Figure S8C). Together, these results suggest a potential functional role for the lncRNAs identified through our analysis in macrophages.

### 2.3. SUGCT-AS1-Depleted THP-1 Cells Induce a Pathogenic Phenotype in Vascular Smooth Muscle Cells

To investigate whether the effect of macrophages on vascular smooth muscle cells is altered through the regulation of *SUGCT-AS1* in macrophages, we applied conditioned media (CM) from macrophages to vascular smooth muscle cells. Before the *SUGCT-AS1* functional test, the CM treatment experiment was optimized by establishing four different treatment conditions: ctrl (the media without treatment), LPS/IFN-γ (LPS and IFN-γ-added media incubated for one day without cells), M0-CM (the media cultured with M0 macrophages for one day), and M1-CM (the media cultured with M1 macrophages for one day). We applied these media to HCASMCs and incubated them for one day (Figure S9). As a result, it was confirmed that *TAGLN*, *CNN1*, and *ACTA2*, markers for smooth muscle cell contractility, were decreased, and proinflammatory genes (*IL1B*, *IL6*, and *PTGS2*) were increased in cells treated with M1-CM, demonstrating that soluble factors secreted by M1 macrophages can induce a pathogenic phenotype in vascular smooth muscle cells (Figure S9).

**Figure 3:**
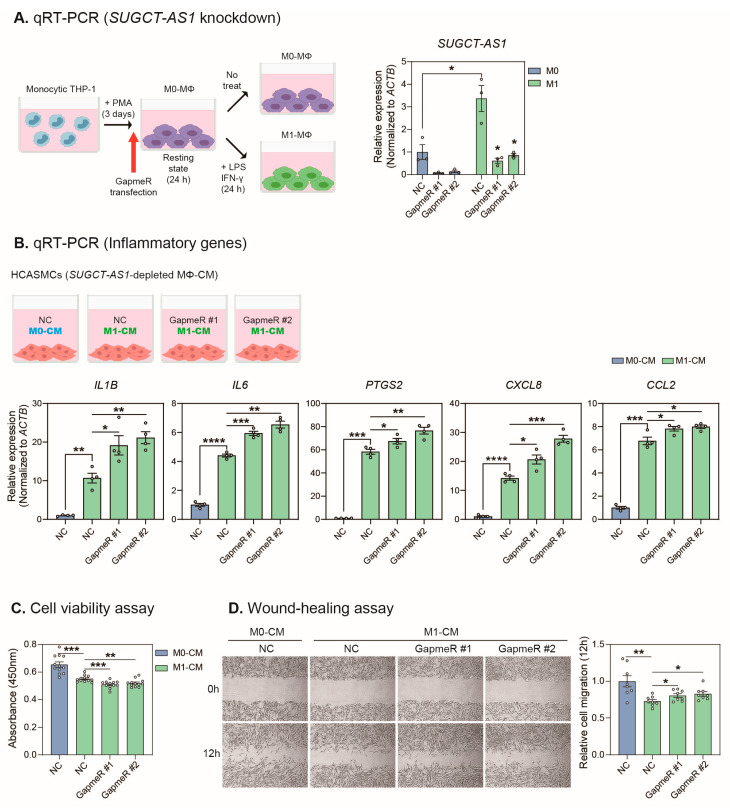
Effect of conditioned media of *SUGCT-AS1*-depleted THP-1 cells on phenotypic changes in vascular smooth muscle cells. (**A**) Experimental design for *SUGCT-AS1* knockdown in M0 and M1 macrophages. Following 24 h of GapmeR transfection, M1 stimulation was performed for 24 h, and depletion of *SUGCT-AS1* was confirmed by qRT-PCR (*n* = 3). NC: negative control GapmeR. (**B**) Top: Conditioned media (CM) of THP-1-derived macrophages (M0 and M1) transfected with GapmeRs (NC, GapmeR #1, and #2) were treated with HCASMCs. Bottom: qRT-PCR measurement of inflammatory genes (*IL1B*, *IL6*, *PTGS2*, *CXCL8*, and *CCL2*) in HCASMCs after 24 h of CM treatment (*n* = 4). The RNA expression was normalized to *ACTB*. (**C**) Cell viability assay in HCASMCs after 24 h of CM treatment (*n* = 12 for each of the transfected samples merged from 3 independent experiments). (**D**) Wound-healing assay in HCASMCs after 0 to 12 h of CM treatment (*n* = 8). The circles in the bars indicate each data point. Data are presented as mean ± SEM. An unpaired two-tailed *t*-test with Welch’s correction was used for statistical analysis. * *p* < 0.05, ** *p* < 0.01, *** *p* < 0.005, **** *p* < 0.001.

To effectively deplete *SUGCT-AS1* located in the nucleus of macrophages, knockdown was performed using a GapmeR antisense oligonucleotide. Monocytic THP-1-derived M0 macrophages were transfected with GapmeRs, incubated for one day, and treated with the M1 stimuli for an additional day. We confirmed that GapmeR #1 and GapmeR #2 (each targeting a different region of *SUGCT-AS1*) effectively knocked down *SUGCT-AS1* in M0 and M1 macrophages (Figure 3A). When treated with the CM of *SUGCT-AS1*-depleted M1 macrophages, the expression of inflammatory genes (*IL1B*, *IL6*, *PTGS2*, *CXCL8*, and *CCL2*) in HCASMCs increased significantly compared to the treatment of CM from the control oligonucleotide-treated M1 macrophage, suggesting that *SUGCT-AS1* may function in relation to the secretion of soluble factors, such as cytokines in macrophages (Figure 3B). Moreover, decreased viability and increased migration of HCASMCs were observed (Figure 3C,D), and the expression of contractile marker proteins (ACTA2 and SM22α) was decreased (Figure S10). Taken together, our results showed that changes in the secreted factors of M1 macrophages by *SUGCT-AS1* depletion induced a pathogenic phenotype in vascular smooth muscle cells.

### 2.4. Depletion of SUGCT-AS1 Promotes the Secretion of Proinflammatory Cytokines in THP-1 Cells

**Figure 4:**
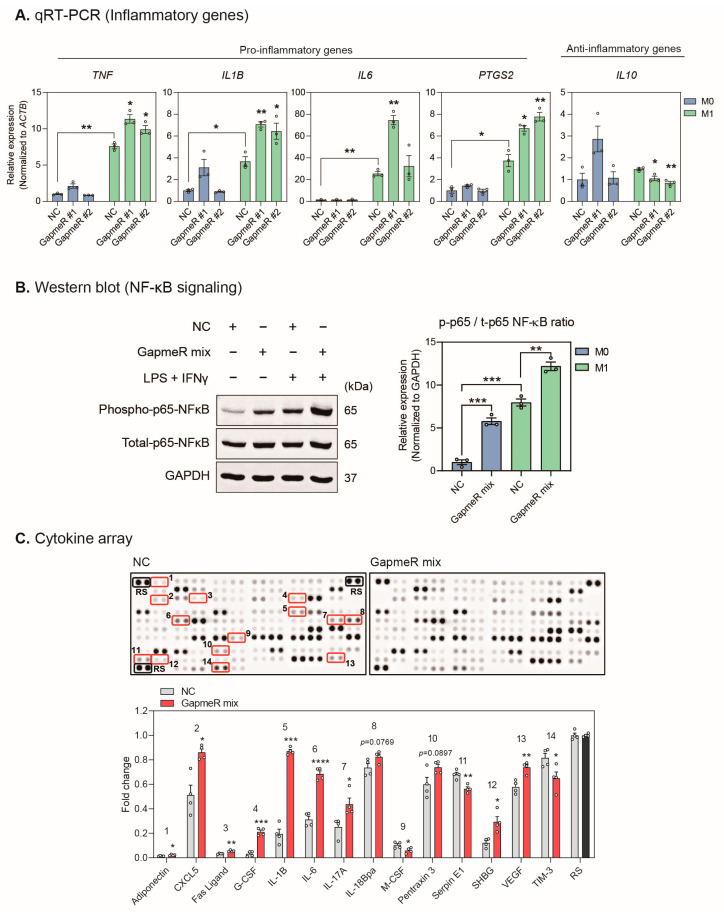
Effect of *SUGCT-AS1* knockdown on proinflammatory cytokine release. (**A**) qRT-PCR measurement of pro- and anti-inflammatory genes (*TNF*, *IL1B*, *IL6*, *PTGS2*, and *IL10*) in THP-1-derived macrophages (M0 and M1) transfected with GapmeRs (NC, GapmeR #1, and #2) (*n* = 3). M1 stimulation was treated for 24 h. The RNA expression was normalized to *ACTB*. (**B**) Western blot analysis of NF-κB p65 activation in THP-1-derived macrophages (M0 and M1) transfected with GapmeRs (NC and GapmeR mix) (*n* = 3). M1 stimulation was performed for 30 min. The ratio of phosphorylated p65 to total p65 was determined (phospho-p65/t-p65 ratio). The GapmeR mix is a mixture of GapmeR #1 and #2. The protein expression was normalized to GAPDH. (**C**) Human cytokine array in conditioned media (CM) of THP-1-derived M1 macrophages transfected with GapmeRs (NC or GapmeR mix). M1 stimulation was treated for 24 h. The differentially measured spots for cytokines and chemokines are labeled with Arabic numerals and red boxes between NC and GapmeR mix (*n* = 4 cultures per group). Each spot was normalized to reference spots (RS). The circles in the bars indicate each data point. Data are presented as mean ± SEM. An unpaired two-tailed *t*-test with Welch’s correction was used for statistical analysis. * *p* < 0.05, ** *p* < 0.01, *** *p* < 0.005, **** *p* < 0.001.

Next, we investigated whether *SUGCT-AS1* modulates inflammatory genes in macrophages. When *SUGCT-AS1* was depleted in M0 and M1 macrophages, the expression of proinflammatory genes (*TNF*, *IL1B*, *IL6*, and *PTGS2*) was significantly increased, and the anti-inflammatory gene *IL10* was decreased in M1 macrophages (Figure 4A). These results indicate that *SUGCT-AS1* acts to suppress the proinflammatory function of M1 macrophages. In addition, it was also confirmed that the p65 subunit of NF-κB, an early key signaling pathway for proinflammatory gene expression, was activated by *SUGCT-AS1* depletion (Figure 4B and Figure S11). Therefore, we analyzed the conditioned media of *SUGCT-AS1*-depleted M1 macrophages to check whether *SUGCT-AS1* affects the cytokine secretion of macrophages. From the result of performing the human cytokine array, significant changes were detected for 14 factors among various cytokines (Figure 4C). In particular, the levels of proinflammatory factors, including IL-1B, IL-6, CXC motif chemokine 5 (CXCL5), and interleukin-17A (IL-17A), markedly increased. Thus, our results demonstrated that *SUGCT-AS1*-depleted M1 macrophages induce inflammatory and pathogenic phenotypes in vascular smooth muscle cells by promoting the secretion of proinflammatory factors.

### 2.5. SUGCT-AS1 Alters the Expression of Genes Involved in Inflammation and the Response to Cytokines

**Figure 5:**
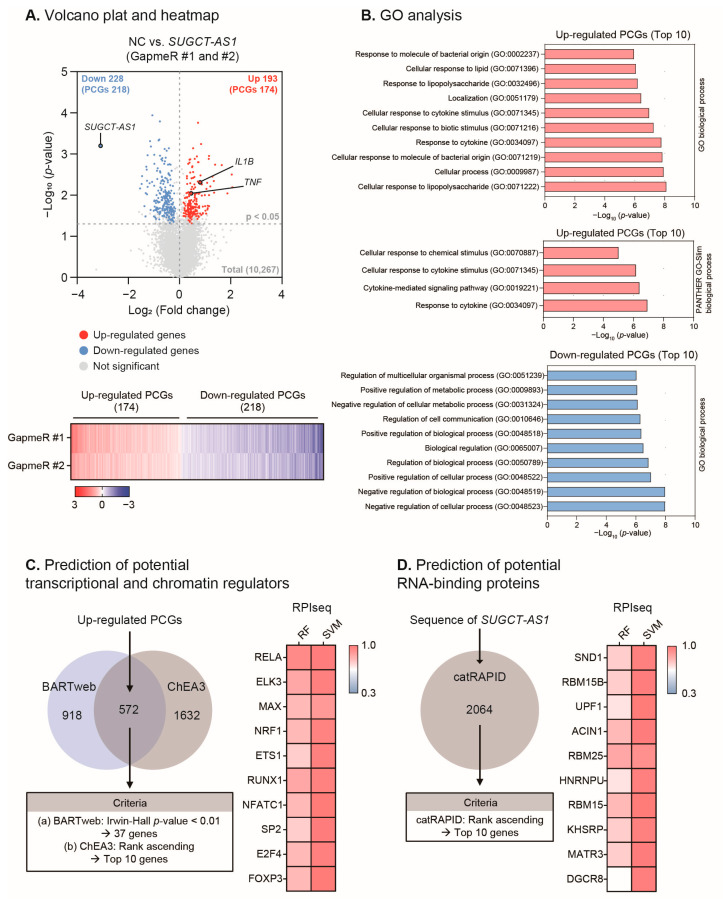
Transcriptomic and bioinformatic analysis of *SUGCT-AS1*-depleted cells. (**A**) Differentially expressed genes in response to *SUGCT-AS1* knockdown in THP-1-derived M1 macrophages stimulated for 24 h. For those genes with statistically significant expression change (*p*-value < 0.05) in the GapmeR-treated samples compared to the control-treated (NC) sample, and those with common expression changes in both GapmeRs (GapmeR #1 and #2), the upregulated genes were indicated with red dots, while downregulated genes were indicated with blue dots, respectively. Note that the gray dots above the *p*-value = 0.05 line represent those genes whose expression change is significant only in one of the two GapmeR-treated samples. (**B**) The Gene Ontology (GO) analysis of differentially expressed protein-coding genes (PCGs) from the gene sets in (**A**). (Top and Middle: analysis for 174 upregulated PCGs; Bottom: analysis for 218 downregulated PCGs). Based on the false discovery rate (FDR) *q*-value, up to the top 10 GO terms were shown. (**C**) Prediction of potential transcriptional and chromatin regulators regulating the transcription of PCGs upregulated by *SUGCT-AS1* depletion from RNA-seq data. Left: Based on BARTweb’s Irwin–Hall *p*-value and ChEA3′s rank, 37 common proteins were selected. Right: The probability of interaction between *SUGCT-AS1* and the top 10 common proteins among these 37 was predicted by RPIseq. The heatmap represents interaction scores from 0 to 1 (RF: random forest, SVM: support vector forest). (**D**) Prediction of potential RNA-binding proteins (RBPs) capable of binding to *SUGCT-AS1*. Left: based on catRAPID’s rank, the top 10 RBPs were selected. Right: the probability of interaction between *SUGCT-AS1* and the top 10 RBPs was predicted by RPIseq.

To gain more insight into the molecular mechanisms of the function of *SUGCT-AS1* in macrophages, we analyzed transcriptome changes in response to *SUGCT-AS1* depletion using RNA-seq. First, we knocked down *SUGCT-AS1* using two GapmeR in M1 macrophages and performed RNA-seq. Among the total 10,267 genes identified, 421 genes (193 increased genes and 228 decreased genes) showed the same expression pattern and statistical significance in both GapmeR treatment groups (Figure 5A). *SUGCT-AS1* was the most downregulated gene following the knockdown, while *IL1B* and *TNF* were upregulated, as shown in Figure 4A, indicating that *SUGCT-AS1* knockdown for the RNA sequencing analysis was performed properly. *SUGCT*, the host gene of *SUGCT-AS1*, was not affected by *SUGCT-AS1* depletion, indicating a low probability of cis-acting regulation of *SUGCT-AS1* (Figure S12). Among these significantly changed genes, 392 genes (174 increased genes and 218 decreased genes) were identified as protein-coding genes (PCGs), and these PCGs were evaluated for common biological pathways by Gene Ontology (GO) analysis (Figure 5B). The top 10 GO terms enriched for the 174 upregulated PCGs were predominantly related to “cytokine” in the biological process, demonstrating that *SUGCT-AS1* modulates cytokine-related inflammatory pathways. Conversely, analysis of the 218 downregulated genes identified relatively comprehensive GO terms, such as “cellular” and “biological” processes or “metabolic” process.

Based on these results, we conducted further analysis to elucidate a functional interaction network involving *SUGCT-AS1,* focusing on the upregulated PCGs. To predict the upstream factors, such as transcription and chromatin regulators, which can commonly regulate these upregulated PCGs, we utilized the public prediction tools BARTweb and ChEA3 (Figure 5C and see Section 4). Based on the prediction scores for each of the two tools, a comparative analysis was performed to select the top 10 genes. For each of these genes, we evaluated their potential to bind to *SUGCT-AS1* using RPIseq, an RNA–protein interaction prediction tool. This analysis revealed that the top 10 transcriptional and chromatin regulators, which included RELA (NF-κB p65 subunit), ELK3 (ETS domain-containing protein), MAX (MYC-associated factor X), NRF1 (nuclear respiratory factor 1), ETS1 (ETS proto-oncogene 1), RUNX1 (runt-related transcription factor 1), NFATC1 (nuclear factor of activated T-cells, cytoplasmic 1), SP2 (Sp2 transcription factor), E2F4 (E2F transcription factor 4), and FOXP3 (forkhead box P3), all possessed a high potential of binding to *SUGCT-AS1*.

In another analysis, we predicted proteins capable of binding to *SUGCT-AS1* using catRAPID, a transcript sequence-based RNA-binding protein (RBP) prediction tool (Figure 5D and see Section 4). The top 10 RBPs were selected based on the rank of catRAPID: SND1 (staphylococcal nuclease domain-containing protein 1), RBM15B (RNA binding motif protein 15B), UPF1 (regulator of nonsense transcripts 1, RENT1), ACIN1 (apoptotic chromatin condensation inducer 1), RBM25 (RNA binding motif protein 25), HNRNPU (heterogeneous nuclear ribonucleoprotein U), RBM15 (RNA binding motif protein 15), KHSRP (KH-type splicing regulatory protein), MATR3 (matrin 3), and DGCR8 (DiGeorge syndrome critical region 8). Likewise, the binding potential of these top 10 RBPs to *SUGCT-AS1* was evaluated through RPIseq, which confirmed that they all possessed a high binding probability. Taken together, our analysis predicted that *SUGCT-AS1* may regulate the expression of numerous cytokine-related genes, possibly through various transcriptional regulators or RBPs, suggesting a possible functional network for the mechanism of *SUGCT-AS1*.

### 2.6. SUGCT-AS1 Regulates Alternative Splicing of MALT1 mRNA by Regulating Intracellular Translocation of hnRNPU

**Figure 6:**
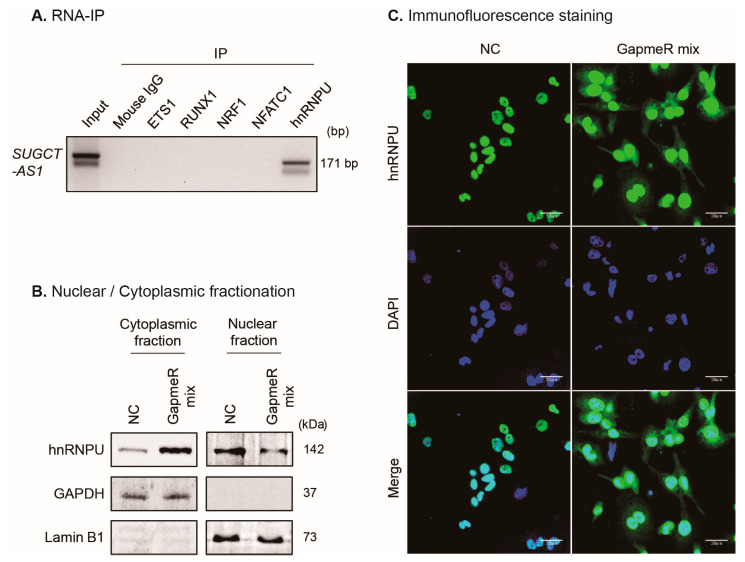
Regulation of nuclear-cytoplasmic translocation of hnRNPU by *SUGCT-AS1*. (**A**) Confirmation of the interactions between *SUGCT-AS1* and predicted proteins (ETS1, RUNX1, NRF1, NFATC1, and hnRNPU) in THP-1-derived M0 macrophages (*n* = 3). IP: Immunoprecipitation. (**B**) Nuclear and cytoplasmic fractionations of proteins in THP-1-derived M0 macrophages transfected with GapmeRs (NC and GapmeR mix) for 24 h (*n* = 3). Lamin B1 was used as a control for nuclear protein, and GAPDH was used as a control for cytoplasmic protein. GapmeR mix is a mixture of GapmeR #1 and #2. (**C**) Immunofluorescence analysis to measure the change in the distribution of hnRNPU after silencing *SUGCT-AS1* for 24 h in THP-1-derived M0 macrophages (*n* = 3). The hnRNPU and nuclei are indicated by green and blue (DAPI), respectively. Scale bars, 30 μm.

To experimentally elucidate the proteins that interact with *SUGCT-AS1* during the regulation of macrophage inflammation, several candidates were selected among the 20 proteins previously listed above. We checked the known function of these proteins and found that the RNA-binding proteins, RBM15, RBM15B, and hnRNPU, alongside the transcriptional and chromatin regulators, ETS1, RUNX1, NRF1, NFATC1, and p65, are identified as being involved in cardiac injury, heart development, cardiovascular disease, and inflammation [28,29,30,31,32,33,34,35,36]. Thus, we verified whether these eight proteins bind to *SUGCT-AS1* by RNA-binding protein immunoprecipitation (RIP). Interestingly, *SUGCT-AS1* bound to hnRNPU and RBM15B, but not to RBM15, ETS1, RUNX1, NRF1, NFATC1, or p65 (Figure 6A and Figure S13A). When we checked the interaction between hnRNPU and RBM15B, they did not bind to each other, suggesting hnRNPU and RBM15B instead independently bind to *SUGCT-AS1* and do not form a complex that binds with *SUGCT-AS1* (Figure S13B).

To check whether these proteins affect the expression of the inflammatory genes, similar to *SUGCT-AS1* depletion (Figure 4A), we designed small interfering RNAs (siRNAs) to suppress their expression. When we performed a knockdown of RBM15B, both siRNAs effectively depleted RBM15B in THP-1-derived macrophages (Figure S14A,B). However, there were no consistent changes in the expression of the proinflammatory genes (*TNF*, *IL1B*, *IL6*, and *PTGS2*) in the same samples (Figure S14C). Therefore, RBM15B was excluded from any subsequent experiments. Another *SUGCT-AS1*-binding protein, hnRNPU, was also effectively depleted at the RNA and protein levels using specific siRNAs (Figure S15A,B). Thereafter, we checked whether its suppression affected the expression of the designated proinflammatory genes. During M1 stimulation, the expression of the inflammatory genes was significantly increased by hnRNPU depletion (Figure S15C). The analysis of gene expression levels in THP-1-derived macrophages showed that both *SUGCT-AS1* (228th highest expression out of 24,881 non-coding RNAs) and *HNRNPU* (899th highest expression out of 20,285 protein-coding genes) are highly abundant in the cells (Figure S16A,B). Taken together, these indicate the possibility that *SUGCT-AS1* is functionally associated with hnRNPU.

To elucidate how *SUGCT-AS1* functions with hnRNPU, we first investigated whether the expression of hnRNPU was altered. However, changes in the mRNA level of hnRNPU were not confirmed in our *SUGCT-AS1*-depleted RNA-seq data (Figure S17A). Its protein level also did not change in *SUGCT-AS1*-depleted Western blot analysis (Figure S17B). Previous reports have shown that most hnRNPs are present in the nucleus, but some are known to translocate between the nucleus and the cytoplasm [37,38]. To investigate the role of *SUGCT-AS1* on the intracellular distribution of hnRNPU, we fractionated THP-1-derived M0 macrophages treated with GapmeRs into the nucleus and cytoplasm. Strikingly, we confirmed that a large fraction of hnRNPU proteins in the nucleus were translocated to the cytoplasm following *SUGCT-AS1* depletion (Figure 6B). In addition, this result was verified through immunofluorescence staining using the hnRNPU antibody (Figure 6C). However, even after GapmeR treatment against *SUGCT-AS1*, the amount of hnRNPU proteins remaining in the nucleus was high, suggesting that it may also be regulated by factors other than *SUGCT-AS1*.

**Figure 7:**
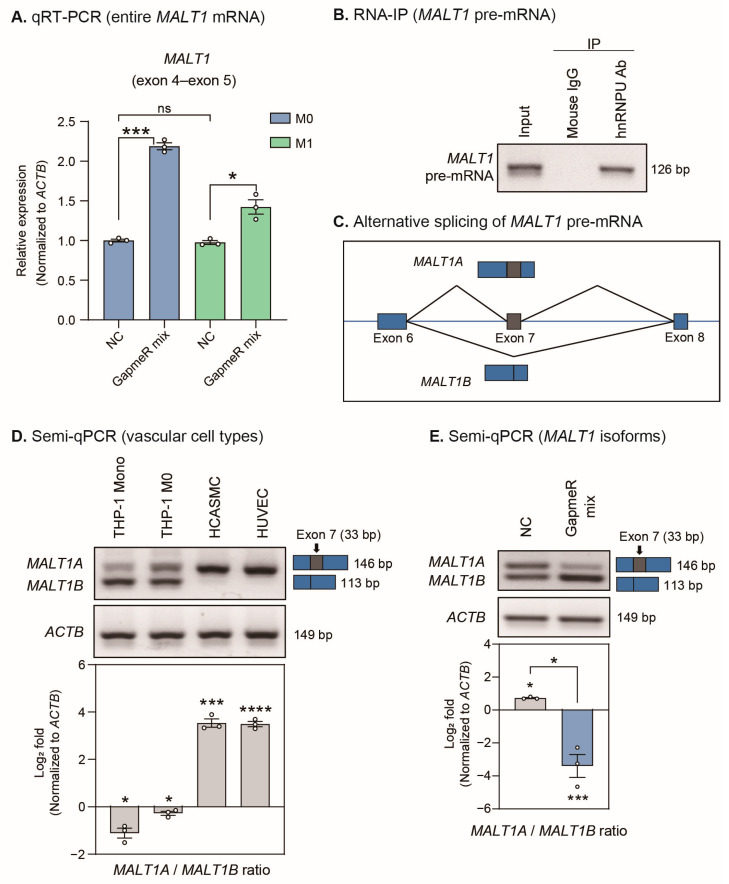
Regulation of alternative splicing in *MALT1* mRNA by *SUGCT-AS1* and hnRNPU. (**A**) qRT-PCR measurement of entire *MALT1* mRNA (exon 4–exon 5) in THP-1-derived macrophages (M0 and M1) transfected with GapmeRs (NC and GapmeR mix) (*n* = 3). M1 stimulation was performed for 24 h. GapmeR mix is a mixture of GapmeR #1 and #2. (**B**) Confirmation of the interaction between *MALT1* precursor mRNA (pre-mRNA) and hnRNPU in THP-1-derived M0 macrophages (*n* = 3). IP: Immunoprecipitation. (**C**) Alternative splicing of *MALT1* pre-mRNA. *MALT1A*: Exon 7 included isoform; *MALT1B*: Exon 7 excluded isoform. (**D**) Semi-qPCR measurement of the ratio of *MALT1* isoforms (*MALT1A* and *MALT1B*) in vascular cell types (*n* = 3). *MALT1A* (upper band) with exon 7 is longer than *MALT1B* (lower band). THP-1_mono: Monocytic THP-1 cells; THP-1_M0: THP-1-derived macrophages; HCASMCs: Human coronary artery smooth muscle cells; HUVECs: Human umbilical vein endothelial cells. (**E**) Semi-qPCR measurement of the ratio of *MALT1* isoforms in THP-1-derived M0 macrophages transfected with GapmeRs (NC and GapmeR mix) (*n* = 3). The RNA expression was normalized to *ACTB*. The circles in the bars indicate each data point. Data are presented as mean ± SEM. An unpaired two-tailed *t*-test with Welch’s correction was used for statistical analysis. * *p* < 0.05, *** *p* < 0.005, **** *p* < 0.001; ns: not significant.

A recent study reported that alternative splicing of mucosa-associated lymphoid tissue lymphoma translocation protein 1 (*MALT1*) is regulated by hnRNPU in T cells [39]. We also noted that MALT1 regulates the activation of NF-κB signaling [40]. Based on these studies, we investigated whether the change in the nuclear-cytoplasmic distribution of hnRNPU by *SUGCT-AS1* regulated the alternative splicing of *MALT1* in macrophages. Interestingly, when our previous vascular smooth muscle cell RNA-seq data and human macrophage public data (GSE101868) were visualized using the sashimi plot of the integrative genomics viewer (IGV) genome browser, exon 7 exclusion of *MALT1* was observed only in macrophages (Figure S18) [41,42,43]. To test the role of *SUGCT-AS1* in the regulation of *MALT1* splicing, we first designed a PCR primer set (detecting exon 4–exon 5 regions) that can measure both isoforms; from this, we found that the expression of entire *MALT1* mRNA was increased when *SUGCT-AS1* was depleted in both the M0 and M1 macrophages (Figure 7A). From the RIP experiment, it was verified that *MALT1* pre-mRNA (intron 6–exon 7–intron 7 region is detected) binds to hnRNPU in the cells that we tested (Figure 7B and Figure S19). Next, we used semi-qPCR to identify the two transcript isoforms of *MALT1* (*MALT1A* and *MALT1B*) produced by alternative splicing (Figure 7C). *MALT1A* was highly expressed in vascular smooth muscle cells and vascular endothelial cells, and *MALT1B* was highly expressed in monocytic THP-1 and M0 macrophages (Figure 7D). These results indicate that alternative splicing of *MALT1* occurs differently in each vascular cell type. Interestingly, when *SUGCT-AS1* was depleted in THP-1-derived M0 macrophages, the proportion of *MALT1B* was markedly increased (Figure 7E). Conversely, when *SUGCT-AS1* was overexpressed in THP-1 cells, the *MALTA* isoform was increased and the expression of proinflammatory genes was also decreased (Figure S20A,B). Importantly, a recent study showed that selective disruption of the *MALT1B* isoform led to the suppression of NF-κB activation and manifested in severe immunopathology as a symptom of autoimmunity [44]. Overall, our findings suggest that *SUGCT-AS1* depletion induces a change in the intracellular distribution of hnRNPU, thereby affecting the alternative splicing of *MALT1*, which in turn regulates NF-κB signaling in macrophages.

## 3. Discussion

**Figure 8:**
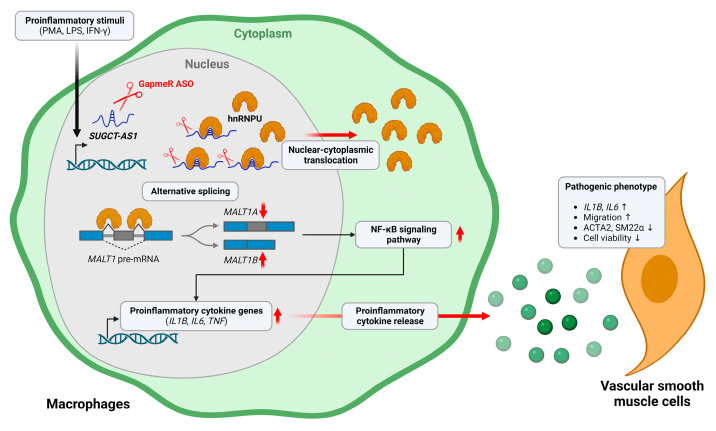
Schematic overview of the role of the lncRNA *SUGCT-AS1* in regulating macrophage inflammation. Depletion of *SUGCT-AS1* by GapmeR antisense oligonucleotides (ASO) in stimulated proinflammatory macrophages induces translocation of hnRNPU protein into the cytoplasm, resulting in increased *MALT1A* isoform and decreased *MALT1B* isoform. This promotes the expression and secretion of cytokines that induce an inflammatory phenotype of vascular smooth muscle cells.

Based on various publicly available RNA sequencing data, our study identified and characterized a novel lncRNA, *SUGCT-AS1*, which is specific to human macrophages, enriched in the nucleus, and upregulated by proinflammatory stimuli. Soluble factors secreted from *SUGCT-AS1*-depleted macrophages further induced the pathogenic phenotype in vascular smooth muscle cells. *SUGCT-AS1* forms a complex with the nuclear protein hnRNPU, which contributes to the nuclear localization of hnRNPU and the alternative splicing of *MALT1*, ultimately affecting NF-κB signaling and the regulation of the expression of proinflammatory genes (Figure 8). Thus, these results suggest that the proinflammatory stimuli-enhanced *SUGCT-AS1* is a repressor with a negative feedback role on the expression of proinflammatory genes. These findings also support the hypothesis that *SUGCT-AS1* is downregulated when atherosclerosis progresses to an unstable stage with various symptoms, including inflammation (Figure 1A and Figure S21).

Our analyses confirmed significant changes in previously reported lncRNAs. *MIR155HG* and *Lnc-DC* were both upregulated in atherosclerotic progression and the M1 macrophage data (Figure S1B). The MIR155 host gene (*MIR155HG*) is a precursor lncRNA encoding microRNA-155 (*miR-155*) [45]. Furthermore, *miR-155* plays a key role in atherosclerosis by regulating the inflammatory response in macrophages and vascular endothelial cells [46,47]. *Lnc-DC* controls human dendritic cell differentiation from monocytes through binding to the transcription factor STAT3 [25,26]. In addition to *SUGCT-AS1*, among other lncRNA candidates, *LINC01176*, *RP11-422J8.1*, and *RP11-1008C21.1* also showed distinct expression changes in M1 or M2 macrophages, suggesting that they may also have macrophage-related functions (Figure 1E and Figure S6B). Another candidate, *DLGAP1-AS1*, was selected through the analysis of atherosclerosis and macrophage data, yet it was also strongly expressed in vascular smooth muscle cells and endothelial cells, indicating the possibility of related pathogenic functions in other vascular cells (Figure S8B). Therefore, our analyses suggest that the lncRNAs identified in this study regulate the inflammation of macrophages and could be involved in pathological processes.

hnRNPU belongs to a subfamily of heterogeneous nuclear ribonucleoproteins (hnRNPs) and has distinct nucleic-acid-binding properties, as it binds to RNA and DNA. A recent study reported that hnRNPU regulates alternative splicing of the *MALT1* paracaspase, a key component of the signaling pathways that mediate the innate and adaptive immune responses [39,48]. MALT1 activates NF-κB signaling by recruiting TNF receptor-associated factor 6 (TRAF6) to its two TRAF6-binding motifs (T6BMs). Moreover, hnRNPU inhibits the production of *MALT1A* by stabilizing the stem-loop RNA structures that maintain the exon 7 skipping of *MALT1*. Accordingly, the downregulation of hnRNPU enhanced the expression of *MALT1A* [48]. However, our results revealed that depletion of *SUGCT-AS1* induced the translocation of hnRNPU to the cytoplasm, resulting in increased *MALT1B* expression (Figure 6B,C and Figure 7E). To date, intracellular translocation of hnRNPU by external stimulation has been reported in some studies; however, its effect on alternative splicing of *MALT1* has not been reported [49,50]. In addition, alternative splicing of *MALT1* determines the presence or absence of a TRAF6 binding motif 1 (T6BM1) on exon 7, although *MALT1B* lacking T6BM1 also showed high sensitivity to NF-κB signaling activity [44]. This supports our results, showing that the enhancement of *MALT1B* expression and NF-κB signaling activity was induced by *SUGCT-AS1* depletion (Figure 4B and Figure 7E).

In the study of lncRNA, the mechanism of gene expression regulation through direct transcription factor regulation has been extensively studied and follows recent studies that have revealed various mechanisms, including epigenetic, epitranscriptomic, and scaffolding [15,51,52]. Although RBM15B was excluded as a candidate due to the inconsistent changes in the expression of proinflammatory genes from the knockdown experiment, it is expected to have other unknown functions because it also binds to *SUGCT-AS1* (Figure S14). RBM15B is a methyltransferase involved in N6-methyladenosine (m^6^A), the most abundant and widespread epitranscriptomic modification of mRNA in mammals. Recent studies have shown that m^6^A modification plays an important role in the risk mechanisms of cardiovascular diseases, such as obesity, inflammation, and hypertension [53]. Additionally, regarding the protein candidates (RBPs, transcriptional and chromatin regulators) that were excluded through the RIP experiments because they did not demonstrate any ability to bind to *SUGCT-AS1* (Figure 6A and Figure S13A), numerous biological mechanism studies have previously been reported on their involvement throughout the cardiovascular system, including in cardiovascular disease, inflammation, and heart development [28,29,31,32,33,34,35,36]. Indeed, RELA (NF-κB p65) did not bind to *SUGCT-AS1* but played an important role, indirectly, through MALT1 processing by hnRNPU (Figure S13A). As such, we anticipate that these other proteins also have additional mechanisms associated with *SUGCT-AS1* in the inflammatory response in macrophages.

For decades, researchers have conducted various studies on cardiovascular diseases, which has resulted in numerous RNA sequencing data being accumulated in public databases. Our research compared and analyzed these RNA sequencing data to identify and validate the *SUGCT-AS1*/hnRNPU complex that regulates NF-κB signaling and inflammation by affecting alternative splicing of *MALT1*. Based on the results that *SUGCT-AS1* depletion enhances the proinflammatory response in macrophages, we suggest that it has the potential to be used as a diagnostic target in various inflammatory diseases.

## 4. Materials and Methods

### 4.1. Selection of lncRNAs Involved in Macrophage Polarization

We obtained four RNA-seq datasets from the Gene Expression Omnibus (GEO) database. These data included RNA-seq data from unstable or stable atherosclerotic plaques (atherosclerosis progression, GEO: GSE120521) and proinflammatory (M1: LPS and IFN-γ) or anti-inflammatory (M2: IL-4) stimuli-treated human CD14^+^ monocyte-derived macrophages (hMFs, GEO: GSE55536, GSE146028, GSE140026) [19,20,21,22]. In the GSE120521 dataset, the stable and unstable plaques regions were dissected at carotid endarterectomy, and the classification into stable and unstable regions was based on macroscopic appearance. The analysis of the RNA-seq data was performed as described previously [54]. In brief, from the raw sequencing reads, the reads with low quality were removed using Trimmomatic, while the remaining reads were aligned to the human genome (hg19) with STAR aligner [55,56]. The fragments per kilobase of transcript per million mapped reads (FPKM) were calculated with the Cuffnorm algorithm [57]. To identify the lncRNAs with differential expression, the lncRNAs with *p*-values less than 0.2 based on a two-tailed *t*-test were selected. To select the candidate lncRNAs with differential expression during the polarization of hMFs to M1 or M2, we first selected only lncRNAs with an average FPKM value greater than 1, and not 0 in the atherosclerosis progression RNA-seq data. Then, we finally selected lncRNAs that showed the same expression pattern (increased or decreased expression) in at least two of the three hMFs RNA-seq data.

### 4.2. Cell Culture

THP-1 human monocytic cell lines (Korean Cell Line Bank, Seoul, Republic of Korea) were maintained in RPMI-1640 (WELGENE, Gyeongsan, Republic of Korea) supplemented with 10% fetal bovine serum (WELGENE, Gyeongsan, Republic of Korea), 0.05 mM 2-mercaptoethanol (Gibco, Waltham, MA, USA), and 1% antibiotic/antimycotic solution (WELGENE, Gyeongsan, Republic of Korea). THP-1 cells were differentiated into macrophage-like cells (M0 THP-1) by treatment with 100 nM phorbol 12-myristate 13-acetate (PMA, Sigma, St. Louis, MO, USA, P8139) for three days. M0 THP-1 cells were stimulated with 10 ng/mL lipopolysaccharide (LPS, Sigma, St. Louis, MO, USA, L4516) and 30 ng/mL interferon-gamma (IFN-γ, Thermo Fisher Scientific, Waltham, MA, USA, PHC4031), or 20 ng/mL interleukin-4 (IL-4, PEPROTECH, Waltham, MA, USA, 200-04). M0 THP-1 cells were polarized into M1 and M2 THP-1 cells, respectively, through these proinflammatory (LPS and IFN-γ) and anti-inflammatory (IL-4) stimuli. Tumor necrosis factor-alpha (TNF-α, 25 ng/mL, Abcam, Cambridge, UK, ab9756) and interleukin-6 (IL-6, 20 ng/mL, PEPROTECH, Waltham, MA, USA, 216-16) were also used to induce inflammation. Human oxidized low-density lipoprotein (oxLDL, 10–50 µg/mL, Kalen Biomedical, Montgomery Village, MD, USA, 770252) was used to induce THP-1-derived macrophages into foam cells. To measure the cell-type specific expression of lncRNAs, we used THP-1 cells, Jurkat clone E6-1 (T-cell lines, Korean Cell Line Bank, Seoul, Republic of Korea), H9 (T-cell lines, Korean Cell Line Bank, Seoul, Republic of Korea), CCRF-SB (B-cell lines, Korean Cell Line Bank, Seoul, Republic of Korea), IM-9 (B-cell lines, Korean Cell Line Bank, Seoul, Republic of Korea), human coronary artery smooth muscle cells (HCASMCs, Gibco, Waltham, MA, USA), and human umbilical vein endothelial cells (HUVECs, ATCC, Manassas, VA, USA). Jurkat, H9, CCRF-SB, and IM-9 were maintained in RPMI-1640 (WELGENE, Gyeongsan, Republic of Korea) supplemented with 10% fetal bovine serum (WELGENE, Gyeongsan, Republic of Korea) and 1% antibiotic/antimycotic solution (WELGENE, Gyeongsan, Republic of Korea). HCASMCs were maintained in Medium 231 (Gibco, Waltham, MA, USA) supplemented with smooth muscle growth supplement (SMGS, Gibco, Waltham, MA, USA) and 1% antibiotic/antimycotic solution (WELGENE, Gyeongsan, Republic of Korea). HCASMCs between passages 4 and 8 were used throughout this study. HUVECs were maintained in Vascular Cell Basal Medium (ATCC, Manassas, VA, USA) supplemented with Endothelial Cell Growth Kit-VEGF (ATCC, Manassas, VA, USA, PCS-100-041) and 1% antibiotic/antimycotic solution (WELGENE, Gyeongsan, Republic of Korea), according to the ATCC guidelines.

### 4.3. RNA Preparation and PCR

Total RNA was isolated using TRIzol reagent (Invitrogen, Waltham, MA, USA), and then residual DNA was removed using DNase I (Takara, Kusatsu, Japan), according to the manufacturer’s protocol. Total RNA was converted to complementary DNA (cDNA) using a RevertAid reverse transcriptase (Thermo Fisher Scientific, Waltham, MA, USA) and random hexamers (Thermo Fisher Scientific, Waltham, MA, USA) or oligo-d(T) primers (Thermo Fisher Scientific, Waltham, MA, USA), according to the manufacturer’s instructions. Quantitative real-time PCR (qRT-PCR) was performed using the Power SYBR Green PCR master mix (Applied Biosystems, Waltham, MA, USA) and the Rotor-Gene Q real-time PCR system (QIAGEN, Hilden, Germany). A semi-quantitative PCR (semi-qPCR) was performed using the nTaq DNA polymerase (Enzynomics, Daejeon, Republic of Korea) in the Master cycler Nexus X2 (Eppendorf, Hamburg, Germany) and evaluated using 2% agarose gel electrophoresis and ImageJ software (V1.53c). The expression of genes was normalized to the expression of *ACTB*. The PCR primers are listed in Supplementary Table S1.

### 4.4. Cellular Fractionation

To fractionate the unstimulated (M0) and stimulated macrophages (M1 and M2) into nuclear and cytoplasmic fractions, the cells were collected and treated with buffer A (10 mM HEPES (pH 7.9), 10 mM KCl, 0.1 mM EDTA, 1 mM DTT), as previously reported [41]. After a 25 min incubation on ice, 10% Nonidet P-40 (NP-40) was added to a final concentration of 0.25% and incubated for an additional 2 min. After centrifugation, the supernatant was directly used as a cytoplasmic protein fraction, or for the extraction of cytoplasmic RNA using a TRIzol LS reagent (Invitrogen, Waltham, MA, USA). The pellet was resuspended in K100 buffer D (20 mM Tris (pH 8.0), 100 mM KCl, 0.2 mM EDTA) followed by centrifugation to obtain nuclear fraction. This nuclear fraction was treated with TRIzol reagent (Invitrogen, Waltham, MA, USA) to isolate nuclear RNA or sonicated in K100 buffer D to isolate nuclear protein. *MALAT1* and precursor *GAPDH* (pre-*GAPDH*) mRNA were used as controls for the nuclear RNA, and mature *ACTB* and mature *GAPDH* mRNAs were used as cytoplasmic RNA controls. Lamin B1 and GAPDH were used as controls for protein levels in nuclear and cytoplasmic fractions, respectively.

### 4.5. Cell Viability Assay

The viability of HCASMCs was measured using the EZ-Cytox cell viability assay kit (DoGEN, Seoul, Republic of Korea), according to the manufacturer’s instructions. Briefly, HCASMCs (5 × 10^3^) were seeded into a 96-well plate and incubated with a conditioned medium of SUGCT-AS1-depleted THP-1 cells one day later. This conditioned medium was used after diluting with RPMI-1640 (with 10% FBS) in a 1:1 ratio. Cells were then incubated with 10 µL of EZ-Cytox reagent (WST: water-soluble tetrazolium salt) for one hour, and absorbance was measured at 450 nm with a microplate reader (BioTek, Winooski, VT, USA).

### 4.6. Wound Healing Assay

A scratch wound healing assay evaluated the ability of the HCASMCs to migrate. When HCASMCs reached full confluency, the cell layer was scratched with a sterile micropipette tip to create a scratch and then washed with PBS. Next, the cells were incubated with a conditioned media (1:1) of SUGCT-AS1-depleted THP-1 cells for 12 h. The picture of the scratched area was taken using an Eclipse Ts2 microscope (Nikon, Tokyo, Japan) and quantified using ImageJ software (V1.53c).

### 4.7. Western Blot Analysis

The cells were harvested and incubated in ice-cold radioimmunoprecipitation assay buffer (RIPA, Translab, Daejeon, Republic of Korea) for 10 min. Protein extracts were quantified using a bicinchoninic acid (BCA) protein assay kit (Thermo Fisher Scientific, Waltham, MA, USA). Protein (10–20 μg) was loaded on an 8–10% sodium dodecyl sulfate (SDS)-polyacrylamide gel and transferred onto a polyvinylidene fluoride membrane (PVDF, Millipore, San Salvador, El Salvador) previously activated by absolute methanol (Merck, Darmstadt, Germany). The membrane was blocked with 5% bovine serum albumin (BSA, GenDEPOT, Katy, TX, USA) for one hour at room temperature, followed by incubation with primary antibodies (1:1000) overnight at 4 °C. Primary antibodies against ACTA2 (Abcam, Cambridge, UK, ab5694), SM22α (Abcam, Cambridge, UK, ab10135), GAPDH (Santa Cruz, Dallas, TX, USA, sc-32233), NF-κB p65 (Abcam, Cambridge, UK, ab16502), phosphorylated (phosphor-) NF-κB p65 (Cell Signaling Technology, Danvers, MA, USA, 3033s), hnRNPU (Santa Cruz, Dallas, TX, USA, sc-32315), RBM15B (Proteintech, Rosemont, IL, USA, 22249-1-AP), and Lamin B1 (Abcam, Cambridge, UK, ab16048) were used. The membrane was incubated with horseradish peroxidase (HRP)-conjugated secondary antibody (1:5000) for one hour at room temperature and visualized using an enhanced chemiluminescence (ECL) solution (Millipore, San Salvador, El Salvador) and Fusion Solo software (Vilber, V16.12). Protein expression was normalized to the expression of GAPDH.

### 4.8. Human Cytokine Array

Conditioned media of THP-1 cells were used for the human XL Cytokine Array Kit (R&D Systems, Minneapolis, MN, USA, ARY022B), according to the manufacturer’s instructions. Conditioned media were centrifuged to remove particulates. Cytokine membrane was incubated in a blocking solution for one hour at room temperature. Then, conditioned media were diluted in a blocking solution, followed by incubation with the cytokine membrane overnight at 4 °C. The membrane was sequentially incubated in a blocking solution containing the antibody detection cocktail for 1 hour and in a streptavidin–horseradish peroxidase (HRP) solution for 30 min at room temperature. Dot blots were visualized using ECL solution and Fusion Solo software (Vilber, V16.12).

### 4.9. Suppression of lncRNA Expression

We used the Antisense LNA GapmeRs (https://geneglobe.qiagen.com/kr/customize/rna-silencing/antisense-lna-gapmers/ accessed on 25 January 2022) to design GapmeRs against *SUGCT-AS1* and used the siDESIGN Center in horizon discovery (https://horizondiscovery.com/en/ordering-and-calculation-tools/sidesign-center/ accessed on 10 April 2022) and i-Score Designer (https://www.med.nagoya-u.ac.jp/neurogenetics/i_Score/i_score.html/ accessed on 10 April 2022) to design siRNAs against *hnRNPU* and *RBM15B*. Negative control Antisense LNA GapmeR (QIAGEN, Hilden, Germany) and AccuTarget negative control siRNAs (Bioneer, Daejeon, Republic of Korea) were used as negative controls, respectively. The sequences of the GapmeRs and siRNAs used are listed in Supplementary Table S1. For the knockdown experiment, 5 × 10^5^ THP-1 cells per well were seeded in a six-well plate and treated with 100 nM PMA. After three days of PMA treatment, GapmeRs (10 nM) or siRNAs (30 nM) were transfected into the M0 macrophages using Lipofectamine RNAiMAX (Invitrogen, Waltham, MA, USA), according to the manufacturer’s instructions.

### 4.10. RNA Sequencing

Three RNA samples from each of the negative control or the *SUGCT-AS1*-depleted (GapmeR #1 and GapmeR #2, respectively) M1 THP-1 cells previously stimulated for 24 h with LPS and IFN-γ were prepared for RNA sequencing (RNA-seq) analysis. Total RNAs were treated with Dnase I, and their integrity was verified with 2100 Bioanalyzer (Agilent, Santa Clara, CA, USA). The RNA-seq libraries were prepared with TruSeq Stranded Total RNA kit (Illumina, San Diego, CA, USA), and NovaSeq 6000 System (Illumina, San Diego, CA, USA) was used to read the sequences. The obtained raw sequence reads were analyzed in the same manner as the analysis of the public data above. From the expression values, those genes with significant expression change (*p*-value < 0.05) in the GapmeR-treated samples compared to the control-treated (NC) sample, and those with common expression changes in both two GapmeRs (GapmeR #1 and #2), were selected.

### 4.11. Bioinformatics Analysis

InteractiVenn (http://www.interactivenn.net/ accessed on 2 January 2022) was used to obtain intersections among gene sets from public RNA-seq data via Venn diagrams. The coding potential calculator 2 (CPC 2.0, http://cpc2.gao-lab.org/ accessed on 18 January 2022) and the coding potential assessment tool (CPAT, http://lilab.research.bcm.edu/ accessed on 18 January 2022) were used to assess the coding potential of the lncRNA transcripts.

Gene Ontology Resource (http://geneontology.org/ accessed on 8 March 2022) was used for gene ontology (GO) enrichment analysis of the selected genes obtained from the RNA-seq of *SUGCT-AS1*-depleted M1 THP-1 cells. Protein-coding genes (PCGs), whose expression levels increased (174 genes) or decreased (218 genes) in response to *SUGCT-AS1* knockdown were applied to the analysis for “GO biological process” and “PANTHER GO-Slim biological process” annotation datasets. The gene list was then evaluated using the PANTHER classification system and Fisher’s exact test and corrected by calculating the false discovery rate (FDR).

To identify the transcription factors and chromatin regulators involved in *SUGCT-AS1*-related cytokine regulation, we used BARTweb (http://bartweb.org/ accessed on 25 March 2022) and ChEA3 (https://maayanlab.cloud/chea3/ accessed on 25 March 2022). The list of PCGs, whose expression level increased in response to *SUGCT-AS1* knockdown, was used as input to BARTweb and ChEA3, respectively, and their results were compared. Then, among the factors with an Irwin–Hall *p*-value of less than 0.01 in BARTweb, the top 10 factors were selected based on the rank of ChEA3. To predict RNA-binding proteins (RBPs) capable of binding to *SUGCT-AS1*, the *SUGCT-AS1* sequences were used as input to the catRAPID (http://s.tartaglialab.com/page/catrapid_group/ accessed on 27 March 2022). We then selected the top 10 RBPs based on the rank of catRAPID. RPISeq (http://pridb.gdcb.iastate.edu/RPISeq/ accessed on 27 March 2022) was used to predict the probability that *SUGCT-AS1*-related proteins (top 10 factors and top 10 RBPs) selected through BARTweb, ChEA3, and catRAPID directly bind to *SUGCT-AS1*. The sequences of *SUGCT-AS1* and its related proteins were used as inputs, and the heatmaps were drawn using the binding probabilities scores predicted by the random forest (RF) and support vector machine (SVM) classifier. The sequences of *SUGCT-AS1* and the related proteins used are listed in Supplementary Table S2.

### 4.12. RNA-Binding Protein Immunoprecipitation

RNA-binding protein immunoprecipitation (RIP) was conducted using a Magna RIP kit (Millipore, San Salvador, El Salvador) to identify the proteins bound to *SUGCT-AS1*, according to the manufacturer’s instructions. Briefly, the cells were lysed in RIP lysis buffer containing an Rnase inhibitor and protease inhibitor cocktail. After centrifugation, the cell lysates were incubated with antibody-conjugated magnetic beads overnight at 4 °C. These beads were treated with primary antibodies against ETS1 (Santa Cruz, Dallas, TX, USA, sc-55581), RUNX1 (Santa Cruz, Dallas, TX, USA, sc-365644), NRF1 (Santa Cruz, Dallas, TX, USA, sc-101102), NFATC1 (Santa Cruz, Dallas, TX, USA, sc-7294), hnRNPU (Santa Cruz, Dallas, TX, USA, sc-32315), RBM15 (Abcam, Cambridge, UK, ab70549), RBM15B (Proteintech, Rosemont, IL, USA, 22249-1-AP), and NF-κB p65 (Abcam, Cambridge, UK, ab16502). To digest the protein, the immunoprecipitates were then shaken in proteinase K buffer containing 10% SDS at 55 °C for 30 min, and RNA was extracted using phenol–chloroform–isoamyl alcohol (125:24:1 (pH 4.3), Thermo Fisher Scientific, Waltham, MA, USA) and chloroform (Thermo Fisher Scientific, Waltham, MA, USA), followed by precipitation with ethanol. This purified RNA was synthesized into cDNA and applied for semi-quantitative PCR.

### 4.13. Immunofluorescence Staining

For the immunofluorescence staining, 2 × 10^5^ THP-1 cells were plated on 18 mm coverslips and incubated with 100 mM PMA for 3 days, followed by fixation in 4% paraformaldehyde solution for 25 min at 4 °C. The cells were then rinsed with PBS three times and incubated with anti-hnRNPU antibody (1:200, Proteintech, Rosemont, IL, USA, 14599-1-AP) in gelatin-blocking buffer (0.1% gelatin, 0.3% Triton X-100, 16 mM sodium phosphate, and 450 mM NaCl (pH 7.4)) overnight at 4 °C. The next day, the cells were rinsed with PBS three times and incubated with the Alexa 488-conjugated anti-rabbit secondary antibody (1:200, Invitrogen, Waltham, MA, USA) for one hour at room temperature. Cell nuclei were counterstained and mounted using a mounting medium containing 4′,6′-diamidino-2-phenylindole (DAPI, Thermo Fisher Scientific, Waltham, MA, USA). Images were captured using the K1-Fluo confocal laser scanning microscope (Nanoscope Systems, Daejeon, Republic of Korea).

### 4.14. Plasmid Construction

The pcDNA3 was used to construct a plasmid containing the *SUGCT-AS1* sequence. The *SUGCT-AS1* sequence was amplified from the cDNA of THP-1 cells through PCR (Figure S3), followed by TA cloning (TOPcloner PCR cloning kit, Enzynomics, Daejeon, Republic of Korea) and sub-cloning into pcDNA3 using KpnI and XhoI restriction enzymes sites (Thermo Fisher Scientific, Waltham, MA, USA). The sequences of the primer sets used are listed in Supplementary Table S1. For overexpression of *SUGCT-AS1*, 1 × 10^6^ THP-1 cells per well were seeded in a six-well plate and treated with 100 nM PMA. After two days of PMA treatment, 1 µg of pcDNA3-*SUGCT-AS1* (pSUGCT-AS1) was transfected into M0 macrophages using Lipofectamine 3000 (Invitrogen, Waltham, MA, USA), according to the manufacturer’s instructions.

### 4.15. Statistical Analysis

Statistical analysis was performed using an unpaired two-tailed *t*-test with Welch’s correction. All data are presented as the mean ± SEM. * *p* < 0.05, ** *p* < 0.01, *** *p* < 0.005, and **** *p* < 0.001 were considered statistically significant. Prism 8 (GraphPad) was used for statistical analyses.

## Data Availability

Publicly available datasets were analyzed in this study. These data can be found here: (https://www.ncbi.nlm.nih.gov/geo/, accession numbers: GSE120521, GSE55536, GSE146028, GSE140026, GSE101868).

## Supplementary Materials

The following supporting information can be downloaded at: https://www.mdpi.com/article/10.3390/ijms241713315/s1.
