# Supplementary material for: Human lncRNA SUGCT-AS1 Regulates the Proinflammatory Response of Macrophage
Source: Int J Mol Sci. 2023 Aug 28;24(17):13315. doi: 10.3390/ijms241713315 (PMC10487982; doi:10.3390/ijms241713315)
Supplement: Supplementary file 1 [file ijms-24-13315-s001.zip › ijms-2514456-supplementary.pdf]

International Journal of *Molecular Sciences*

MDPI

Supplementary Material

Human lncRNA *SUGCT-AS1* regulates the proinflammatory response of macrophage

Yeong-Hwan Lim, Gwangho Yoon, Yeongseo Ryu, Dahee Jeong, Juhyun Song, Yong Sook Kim, Youngkeun Ahn, Hyun Kook, Young-Kook Kim*

* Corresponding Author: Young-Kook Kim; Email: ykk@jnu.ac.kr

1. Supplementary Tables

Table S1. The sequences of PCR primer, siRNA, and GapmeR.

Human PCR primers.

Gene	Forward (5' -> 3')	Reverse (5' -> 3')	Amplicon size (bp)
ACTB	ACTCTTCCAGCCTTCCTTCC	CAATGCCAGGGTACATGGTG	149
GAPDH	CCCTGGCCAAGGTCATCCAT	GGGCCATCCACAGTCTTCTG	97
RPPH1	GCTTGGAACAGACTCACGG	TCTGAATTGGGTTATGAGGTCC	84
pre-GAPDH	CCGTCTTCCGGAAACCAGAT	CCAGCTACAGAAAGGTCAGC	76
MALAT1	AGACAGCAGCAGACAGGATT	AGCTTCCTTCACCAAATCGC	132
TNF	GAACCCCGAGTGACAAGC	TGGGAGTAGATGAGGTACAGG	166
IL1B	TCTTCGACACATGGGATAACG	ACAAAGGACATGGAGAACACC	178
IL6	GTACATCCTCGACGGCATC	ACCTCAAACTCCAAAAGACCAG	198
PTGS2	CCTTCTGCCTGACACCTTTC	TCTGGTCAATGGAAGCCTGT	197
CXCL10	TCTAAGTGGCATTCAAGGAGTAC	TGGCCTTCGATTCTGGATTC	200
CXCL8	GTGTAAACATGACTTCCAAGCTG	GTCCACTCTCAATCACTCTCAG	183
CCL2	CTGAGACTAACCCAGAAACATCC	TGCACTGAGATCTTCCTATTGG	196
CCR7	GGGAAACCAATGAAAAGCGTG	TGGAGCACAAAGACTCGAAC	139
TLR4	TCTACAAAATCCCCGACAACC	GCTCTGATATGCCCCATCTTC	167
IL10	GAGAACCAAGACCCAGACATC	TCACTCATGGCTTTGTAGATGC	185
CD200R1	CATCGTGGGATTCATTTGGTTG	CAGATGCCTTCACCTTGTTTG	167

MRC1	CATTTGCCAGCGACATAACAG	GAGACCAGATTCCCTCCAAAG	198
CD163	GAGTCCCTTCACCATTACTGTG	GACTTTCACTTCCACTCTCCC	133
TGFB1	AACAATTCCTGGCGATACCTC	GTAGTGAACCCGTTGATGTCC	197
LGALS3	GTGCCTTATAACCTGCCTTTG	AGCGTGGGTTAAAGTGGAAG	142
CD68	TCATTCTTTCACCAGCTGTCC	AGAAGGATCAGGCCGATGA	148
CD14	CAGAGGTTCGGAAGACTTATCG	TTCGGAGAAGTTGCAGACG	145
CD3E	GCCTCTGCCTCTTATCAGTTG	TTTATCATCCTCATCACCGCC	188
CD19	TTCTGCCTGTGTTCCCTTG	CGTTCCCGTACTGGTTCTG	148
TAGLN	GTCCGAACCCAGACACAAGT	CTCATGCCATAGGAAGGACC	98
CNN1	TCAAGGCCATCACCAAGTATG	CTTCTCTGCGTACTTCACTCC	167
ACTA2	GGTGCTGTCTCTCTATGCCT	AAGGAATAGCCACGCTCAGT	186
vWF	TCAAAACAAGGAGCAGGACC	CACGTAAGGAACAGAGACCAG	172
RBM15B	CCTGGTCTCCTACTTGAAACAG	AATGTCCTTAGTGCTGACTGG	150
HNRNPU	AGAGTTTTGCTTTTCTTTGGGC	CCAGCCAATACGAACTTCATG	149
MALT1	CACCTTTGAATTCAGCCAGTG	AGAGACGCCATCAACACTTC	82
MALT1 pre-mRNA	TTTTCTGAAACAAGGAAGAACAGA	GTAGGCTAAAAGCACTCCAC	126
Alternative splicing of MALT1 (exon 7 skip)	CGAGACAGTCAAGATAGCAAGAA	ACCTTGTCCTTCGCCAAAG	146 (113)
SUGCT-AS1 (AC004988.1)	TCTCAGGAAATCAACAGGACTG	GCAAAACTCTTCACTGGCAC	171
SUGCT-AS1 (Cloning)	GAATTATGCTTGAAGAAGGTTAATCT	TGTGCTTTAGGTAGTCCAGTT	1771
SUGCT	TGGTCAAAAGGAAGCAAAACG	CTATTGTGTACCCGAAGGTGG	198
RP11-1008C21.1	AACTCAGAAGCACTTGGTACC	GGGTTGCATTCAATAACTCAGG	183
LL21NC02-1C16.2	TGGTTTCAAGTGGGCAGAG	TGTCAAAGTGGCAGAGTGAG	123
LINC01176	TGGTTTAGCATTATCCCCTTGG	AGAATGAGTCTTCCAGCCTTG	118
RP4-591C20.9	TGTGCAAGTGTATCTGTAGCC	CACTGGCTATCATTCTGTAGGG	122
DLGAP1-AS1	TCTGTACGTTGCAATCTGTGG	AGCATTTATCACACTAGTACCGTG	150
RP11-422J8.1	CTCGGACCTAAGAACTTGACAG	AAGAGCTTGAGATCAGCCTG	119
RP11-6N17.4	AGCTTTTACCTGCTCTTCGG	GCCTCAATCCTTCTACCACAG	79
RP11-206L10.11	TCACAATGGCATGATCTCGG	ACCCTGTGGCACTGAATATTC	148
CD63-AS1	CTATACCTCCGTGCCAACTC	AGCACAGACCTTGTCAATCC	197
RP11-452L6.5	TTTGTCATCTGCCCCTTCC	ATGTTCAGTCCCCAATGTCC	131
RP11-63P12.6	TTGCTTGTCCTGCTAAGACC	CAGCTCTGGATTTACTTCTAGGG	104
RP11-680A11.5	CTGGTGATGGTCATATCGGTG	CGGTGACAAGAGCAGGATT	137
ZBED5-AS1	TTCTCAAACTTCACTAGCCCC	TGCAAATCCAGTGGTCCTAC	180

RP11-4B16.4	TGGATCGTGTCTGCCTAAAAG	GACATGGATGCCTCAGATGA G	141
AC079767.4	CATGAAAGCAAACACTGGGC	GCGGAAACTGTTAGATGCAA G	146
RP11-452F19.3	GCAGACACCATCTTATTTCTG TTG	TGCACTTGTGTAGTCATGGG	142
XXbac-BPG246D15.8	CCAGGTGCAGCTTCAGAG	TGTGTTACCTCCTTTCCAAGC	95
AC093627.10	GGCAGAGGAAGCTGGAAG	CCAGTCCCCATAAATGTGAG AG	87
CH17-340M24.3	GGCCAATCCCTGTTTTAATCC	TGTCATTCCAATCTCTGCCTTC	150
CD27-AS1	TTGCCTGGAACTTACCACTG	AATTTGTCCCTCTCCTTGTCC	145
RP11-344B5.2	TGACTTTGAAGCCCACTGAC	GAAGTTCTTGGACCCCTGG	92

Human siRNAs

**Name**	**Sense (overhang) 5' -> 3'**	**Antisense (overhang) 5' -> 3'**
RBM15B siRNA #1	GGGAAGAAGGCAAGAGACA(UU)	UGUCUCUUGCCUUCUUCCC(UU)
RBM15B siRNA #2	GGGCAAGCUAGAAGAAGAA(UU)	UUCUUCUUCUAGCUUGCCC(UU)
hnRNPU siRNA #1	ACAGAAAGGCGGAGAUAAA(UU)	UUUAUCUCCGCCUUUCUGU(UU)
hnRNPU siRNA #2	GAAGAAAGAUUGUGAAGUU(UU)	AACUUCACAAUCUUUCUUC(UU)

Human antisense LNA GapmeRs

**Name**	**Sequence 5' -> 3'**	**Lot number**
SUGCT-AS1 GapmeR #1	G*A*A*G*G*C*T*G*A*G*C*G*T*C*T*G	434942700
SUGCT-AS1 GapmeR #2	T*T*A*A*C*C*T*T*C*T*T*C*A*A*G*C	436013070
Negative control	A*A*C*A*C*G*T*C*T*A*T*A*C*G*C	10909323-1

Table S2. The sequence of *SUGCT-AS1* and predicted binding proteins

Input: *SUGCT-AS1* sequence (1,771 nucleotides)

GAA TTA TGC TTG AAG AAG GTT AAT CTT ATA ATG GCA TGC AGA GCG GAG CTG GGC AAG GCC AGA GGC
TAA ATA TGG AGC CAG AAA GAT ACT GCA GAA AGT GAG CAA AAA GGA TGA GTG GAT GGC CTG GGG TGG
AAT CCG TGA AAA AGG GCA GAT TGT CAG TCT CAG GAA ATC AAC AGG ACT GAG TTC TAA CTT CAG CTG
GGC TAC TAT GCC CTG GGG AAT CAG AAG ATT CTC CCT AAC ATT GGA GTC CAA AGA AGA GGA GCC ACC
ATC TCC TTA ACT ACA AAA CAA CTG AGA GAA GAA ATG TCT CCC TTT AGT GCC AGT GAA GAG TTT TGC
TTC TCT AGA ACA ACT GTT TAT TTT TGA TCT TAT GAA ACG CGT GAC ACT TGT AAC TGA TTT GCC TTC
TTT TTG CTT GCT GCT AGA AAT CTC AGA ACC AAA TTC GAG GTC ATT TCT GAA TGT GCT GGA CTA CAC
AGC ATG AAT TCT GTG TCC TCT CAC CTC ATT CAT AGA TAC ATT TTT TTA TTC CTA CCC ATC TTT TCC
TTT CCC CAG ATT TTC CTA TTG CTC TTT TAT CAT CTT GAG TAA AGA AGG TTA AAC TAT GAA TAA AAA
GTA CAA TCT CAA TGA CGA GTC AGG GAA AAG TAA GTT TTG GAC TTC CAT TTA GAG CCA TTT ATA GGT
CAG CAA TCT TTT GTC TCT GTG TCT TCT TTC TGC CCT GAA CAA CCA AGG CAT CCT AGT CAG TCT CTC
TTC ATC TGC ATA CCA CTT TAA AGA GCC CAG GGG TCT TCC AAG TAC AGT CAC TAC TAA GAA AAT GTC
TTC AGT AAA TTT TTT TTT TTT TTT TTT GAG ATG GAG TCT CGC TCT GTC GCC CAG GCT GGA ATG TAG
TGG CAC GAT CTC AGC TCA CTG CAA GCT CTG CCT CCC GGG TTC ACG CCA TCC TCC TGC CTC AGC CTC
CCA AGT AGC TGA GAC TAC AGG CAC CTG CCA CCA TGC CCG GCT AAT TTT TTT TGT ATT TTT AGT AGA
GAC GGG GTT TCA CTG TAT TAG CCA GGA TGG TCT TAA TCT CCT GAC CTC ATG ATC CGC CCG CCT CGG
CCT CCC AAA GTG CTG GGA TTA CAG GCG TGA GCC CCC GCG CCC AGC CTT CAG TAA AAT TTT AAT GCA
TGT TGC TTA TCA TTC TTT GTA TTG TAA GCA CCA ATC CTT AAT CTG TGC TGT GGT TTT TGT TCA GAA
ACA TTT CTT TAA AAA AAT TTC CCC AGA CGC TCA GCC TTC ATA TTC TAA TCT TTA TTA TTC TGA ACT
ACC TAC AAA GCT AAA TAT ACC TTG TCT ACA TGC AGT GCT TAT GTA ACA ATG GTG CTT ATT TTG TTT
TAC ATT GCT TCA ACC TCT GAC CAG AAA CTT AGA GGA CAG AAT GCC CTG CTT TTA CAA AAA ATG TGG
TGA CCA CAA CGG TAA CCT TAT CTA ATT GAG ACC ATT TTA GGA AGG AGG GGA GGA GAT ATT TTT ACT
GCA AAG ACT GAG TCA TAA GCC TTC ATT TTT TAC CCT GGG TTG TGC AGA AAC TTT AAA CTT TCT ATA
TTA TTT CAT TTC TTC CAT ACC AGG ACA TTT ATT TCC TAT TCA GTG ACA ATC TTG ATG TCT TCA TCC
TTC AAA CTG CTC TCT AAC ATA GTT TCA AAA TCC AAG CAT TAA ACA TTA TTA ATG CTC AGG GGA TGA
ATA TGT TCA TTA CCT TTA CTG TAG TGA TGA TTT CCT ATG GCA AAA TGT TAA ATG ATG TAA AGA TAA
AAA TAA TTA TCA TAG TTA TTA ATC ATA GCA ATA AAA CTG GAC TAC CTA AAG CAC A

RPIseq prediction: *SUGCT-AS1*-protein interaction

	Protein	Length (amino acid)	RF classifier	SVM classifier
BARTweb and ChEA3 prediction Top 10	RELA	551	0.95	0.989
	ELK3	407	0.85	0.985
	MAX	160	0.8	0.894
	NRF1	503	0.8	0.971
	ETS1	441	0.75	0.981
	RUNX1	480	0.85	0.977
	NFATC1	943	0.8	0.996
	SP2	613	0.75	0.993
	E2F4	413	0.8	0.985
	FOXP3	431	0.8	0.995
catRAPID prediction Top 10	SND1	910	0.75	0.982
	RBM15B	890	0.75	0.99
	UPF1	1129	0.7	0.995
	ACIN1	1341	0.8	0.992
	RBM25	843	0.85	0.929
	HNRNPU	825	0.7	0.984
	RBM15	977	0.8	0.996
	KHSRP	711	0.75	0.983
	MATR3	847	0.75	0.992
	DGCR8	773	0.65	0.988

Supplementary Figures

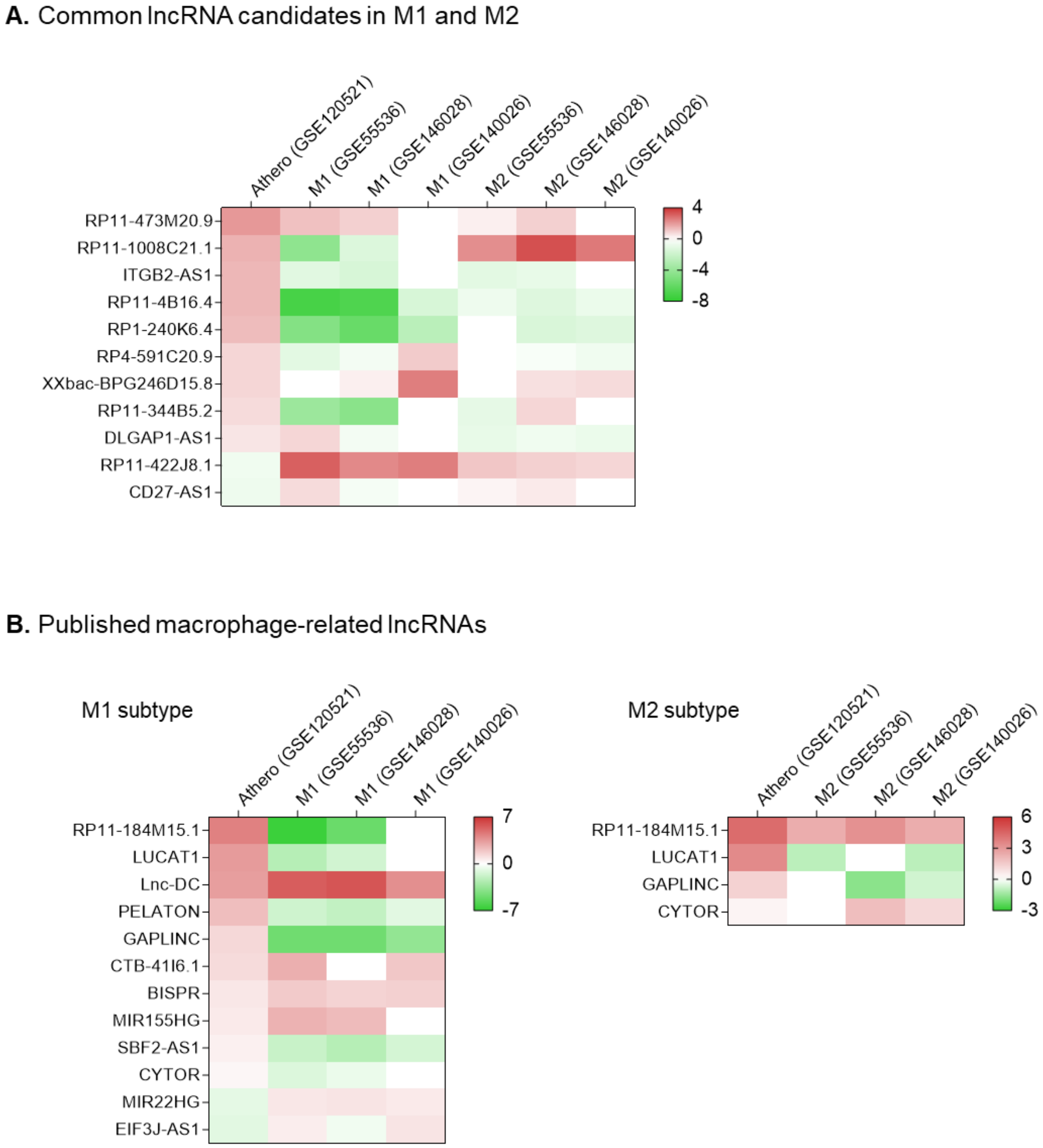

Figure S1. Comparative analysis of the lncRNA expression profiles during atherosclerosis progression and macrophage activation. (A) Atherosclerosis-related lncRNA candidates differentially expressed in both M1 and M2 macrophages. (B) Previously reported lncRNAs differentially expressed in M1 or M2 macrophages. The datasets used for this analysis were obtained from the GEO database (GSE120521, GSE55536, GSE146028, and GSE140026) [19–22].

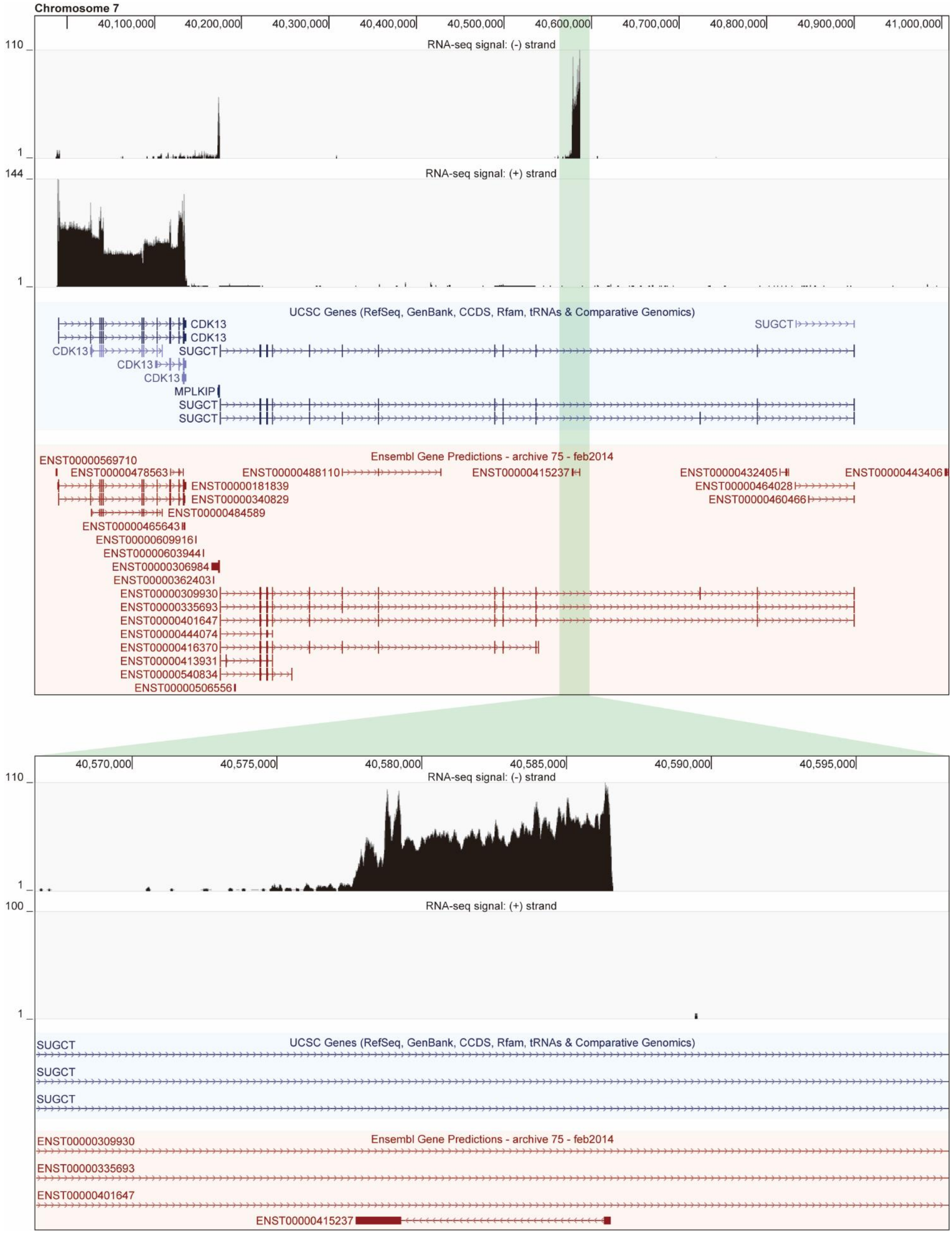

Figure S2. Identification of the transcript structure of *SUGCT-AS1* through the analysis of RNA-seq reads. The sequencing reads were transformed into bedgraph signals and visualized in the UCSC genome browser (*https://genome.ucsc.edu/).*

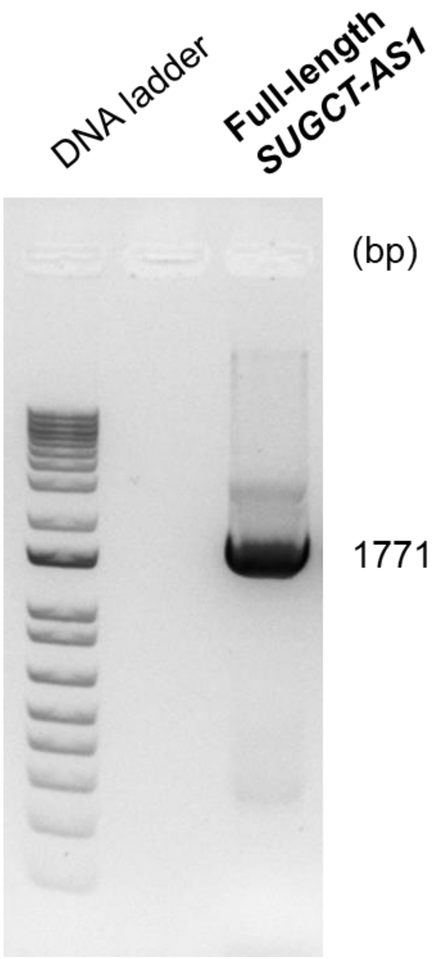

Figure S3. PCR amplification of full-length *SUGCT-AS1*. The PCR primers were designed at both ends of the RNA-seq signals near *SUGCT-AS1* locus as shown in **Figure S2**. The Sanger sequencing of the amplified PCR product confirmed that only one isoform identical to the transcript presents in the database (ENST00000415237; 1,771 base pairs) was detected. The sequences of the primer sets used are listed in **Supplementary Table S1**.

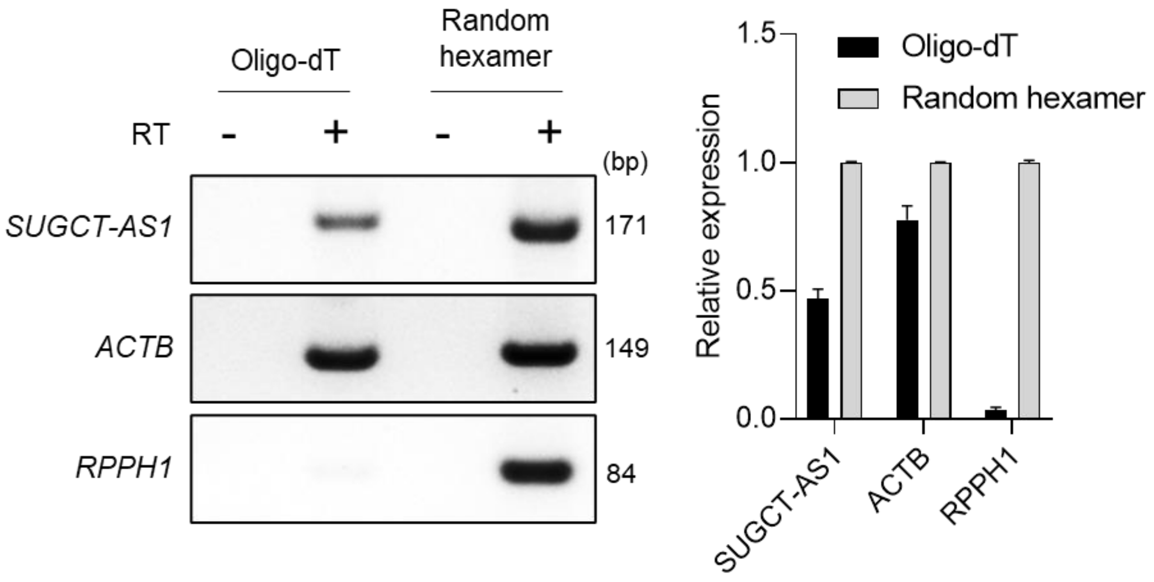

Figure S4. PCR measurement to test the presence of poly(A) tails in *SUGCT-AS1* lncRNA. Oligo-d(T) or random hexamer, respectively, was used as the primer for the reverse transcription. *ACTB* and *RPPH1* (Ribonuclease P RNA Component H1) were used as the controls for RNA with poly(A) tail and that without poly(A) tail, respectively. RT: reverse transcriptase. Data are presented as mean ± SEM ($n = 3$).

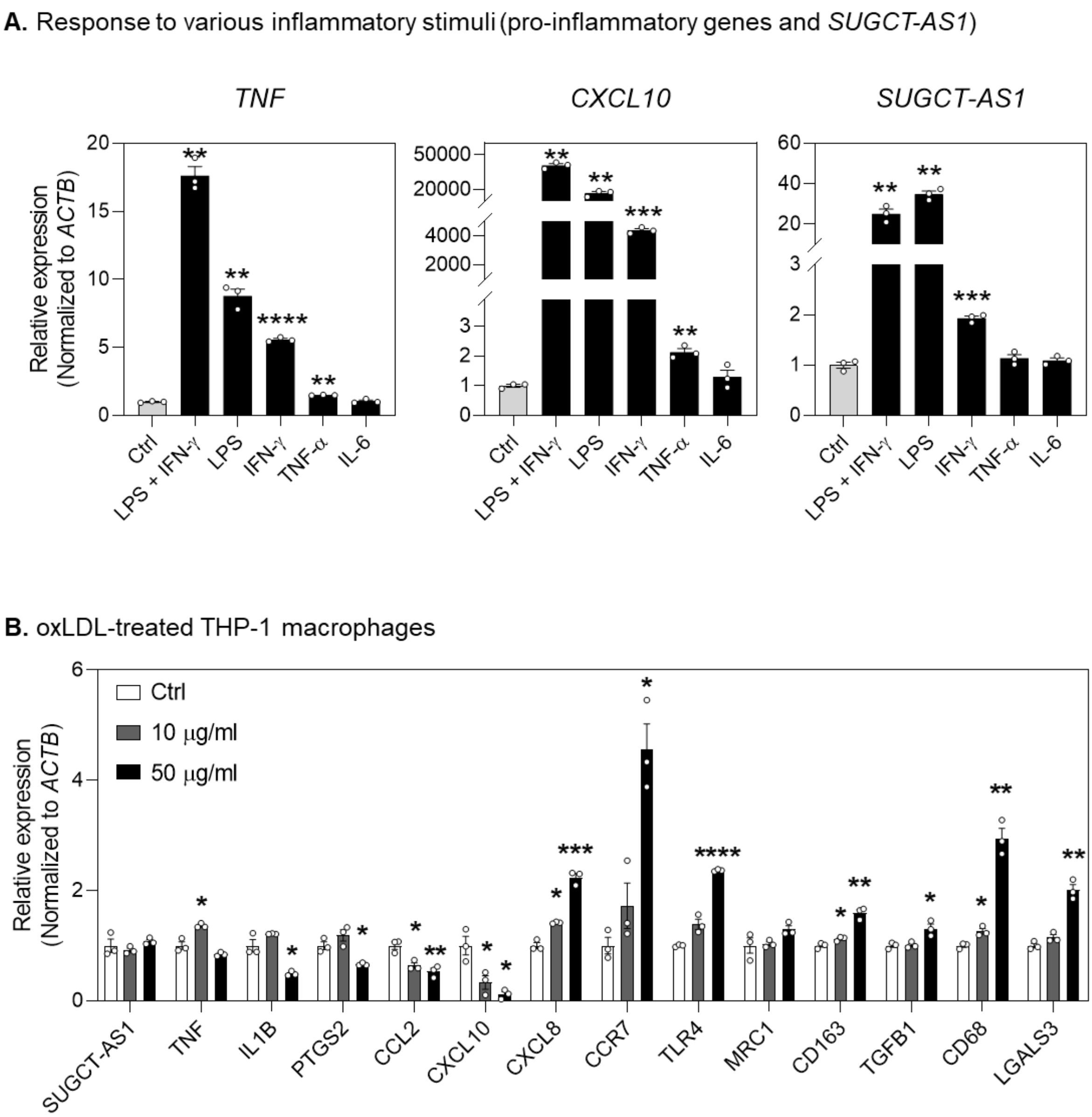

Figure S5. Response of *SUGCT-AS1* to various inflammatory stimuli. qRT-PCR measurement of the expression of (A) inflammatory genes (*TNF* and *CXCL10*) and (B) *SUGCT-AS1* in THP-1-derived macrophages stimulated for 24 hours with various inflammatory stimuli (n = 3). Dosage of inflammatory stimuli: 10 ng/ml LPS, 30 ng/ml IFN-γ, 25 ng/ml TNF-α, and 20 ng/ml IL-6. qRT-PCR measurement of the expression of SUGCT-AS1 and oxidized low-density lipoprotein (oxLDL)-related genes in THP-1-derived macrophages stimulated for 24 hours with oxLDL (10-50 μg/ml, n = 3). The RNA expression was normalized to *ACTB*. Data are presented as mean ± SEM. An unpaired two-tailed t-test with Welch's correction was used for statistical analysis. *$p < 0.05$, **$p < 0.01$, ***$p < 0.005$, ****$p < 0.001$.

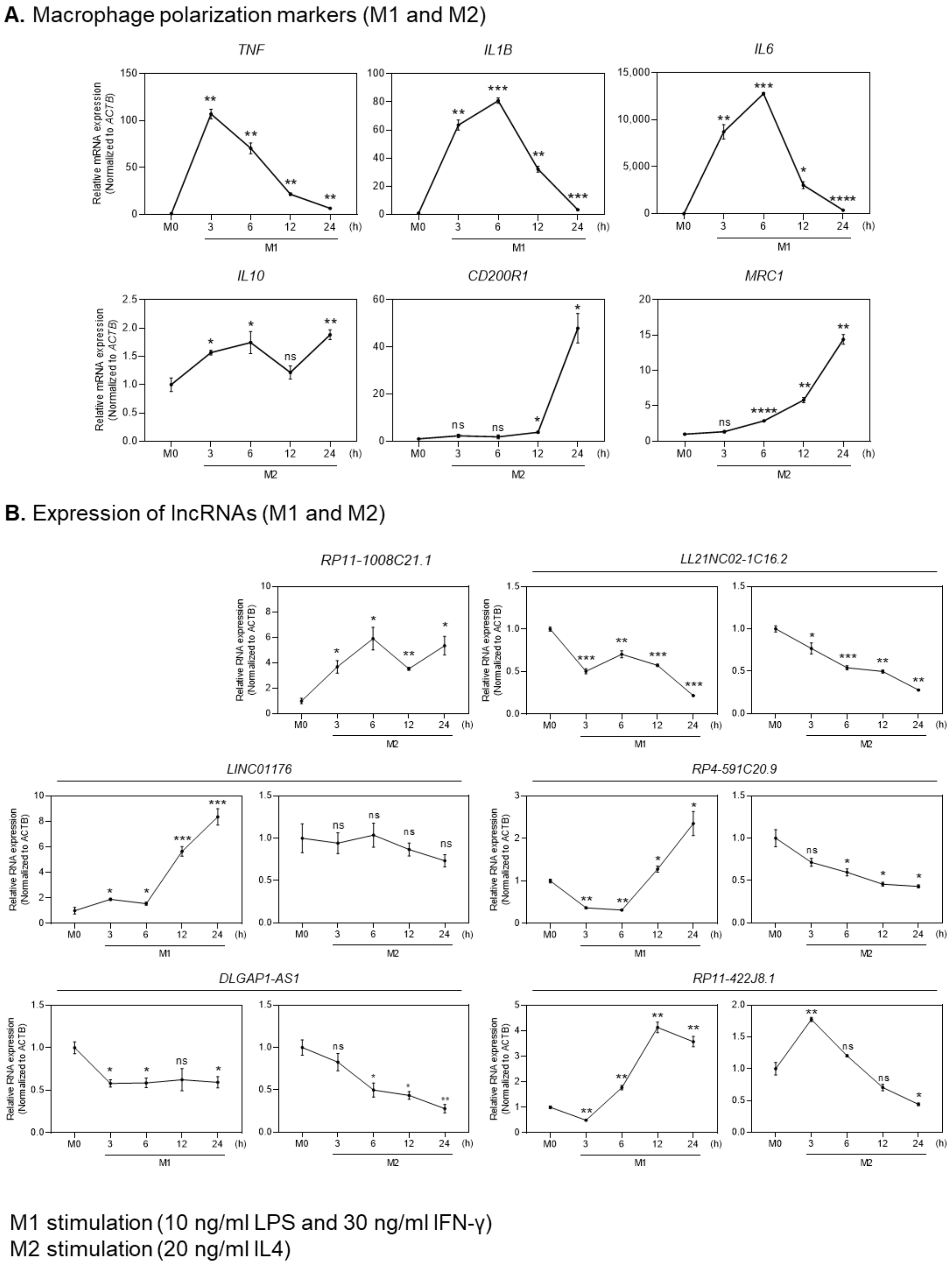

Figure S6. Expression patterns of inflammatory genes and lncRNA candidates depending on the stimulation time. (A) qRT-PCR measurement of marker genes of macrophage polarization in THP-1-derived macrophages treated with stimuli (M1: 10 ng/ml LPS and 30 ng/ml IFN-γ, M2: 20 ng/ml IL-4) at different treatment times ($n = 3$). Proinflammatory (*TNF*, *IL1B*, and *IL6*) and anti-inflammatory (*IL10*, *MRC1*, and *CD200R1*) genes were used as M1 and M2 macrophage markers, respectively. THP-1-derived M0 macrophages were used as controls. (B) qRT-PCR measurement of lncRNA candidates in THP-1-derived macrophages treated with stimuli at different treatment times ($n = 3$). The RNA expression was normalized to *ACTB*. Data are presented as mean ± SEM. An unpaired two-tailed *t*-test with Welch's correction was used for statistical analysis. *$p < 0.05$, **$p < 0.01$, ***$p < 0.005$, ****$p < 0.001$; ns: not significant

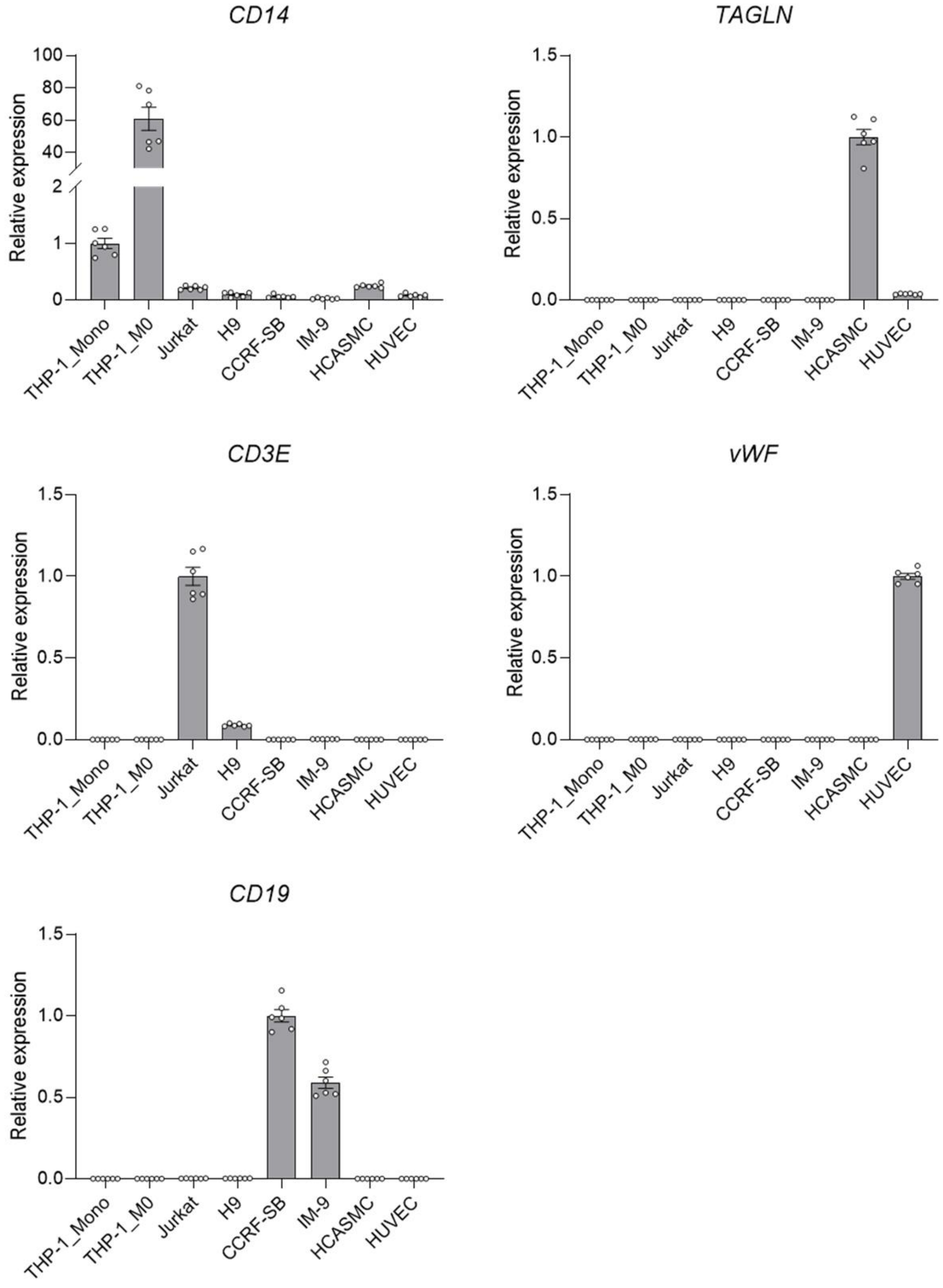

Figure S7. Vascular cell type-specific gene expression. qRT-PCR measurement of vascular cell type-specific expression of markers for each cell type ($n = 6$). THP-1_mono: monocytic THP-1 cells, THP-1_M0: THP-1-derived macrophages, Jurkat and H9: T-cell lines, IM-9 and CCRF-SB: B-cell lines, HCASMCs: human coronary artery smooth muscle cells, HUVECs: human umbilical vein endothelial cells. After normalizing the expression level of each marker gene to that of *ACTB*, their relative expression levels among the cells were compared. The marker gene information is *CD14* for monocyte (THP-1_Mono), *CD3E* for T-cell (Jurkat), *CD19* for B-cell (CCRF-SB), *TAGLN* for smooth muscle cell (HCASMC), and *vWF* for endothelial cell (HUVEC). Data are presented as mean ± SEM.

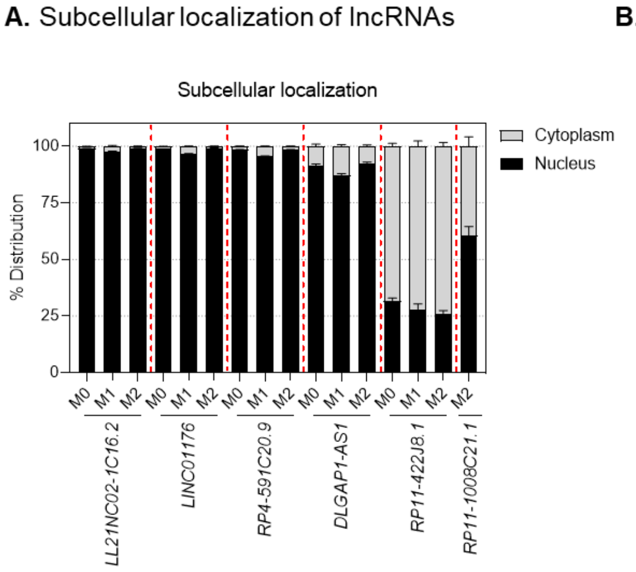

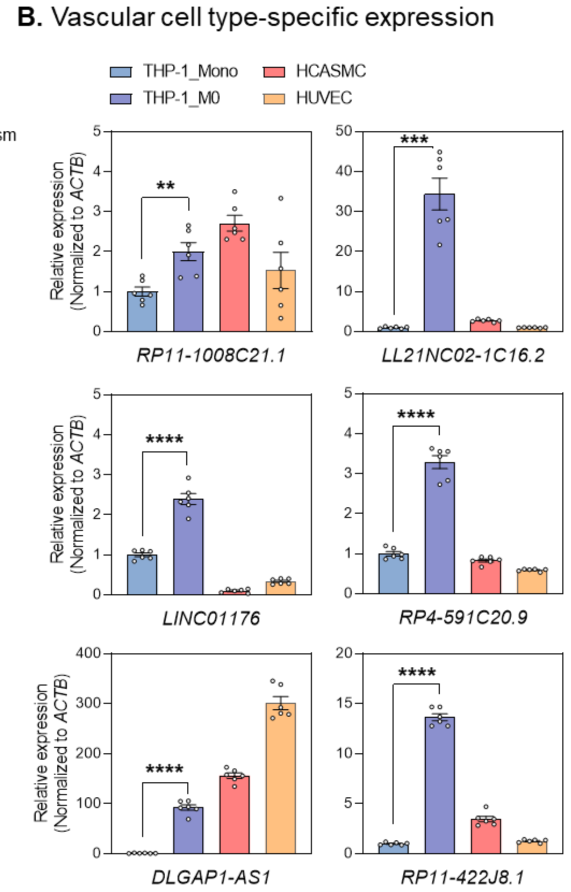

C. Protein coding potential

Gene id	Transcript id	Name	CPAT		CPC2	
			Coding Probability	Label	Coding Probability	Label
ENSG00000259225	ENST00000557883	***RP11-1008C21.1***	0.03805	**no**	0.01508	**noncoding**
ENSG00000272825	ENST00000609953	***LL21NC02-1C16.2***	0.00301	**no**	0.05125	**noncoding**
ENSG00000281404	ENST00000628121	***LINC01176***	0.04743	**no**	0.46465	**noncoding**
ENSG00000268858	ENST00000601296	***RP4-591C20.9***	0.01387	**no**	0.33880	**noncoding**
ENSG00000177337	ENST00000317114	***DLGAP1-AS1***	0.06284	**no**	0.24022	**noncoding**
ENSG00000233621	ENST00000424989	***RP11-422J8.1***	0.01945	**no**	0.05173	**noncoding**

Figure S8. Characterization of lncRNA candidates involved in the differentiation and polarization of macrophages. (A) Subcellular localization of lncRNA candidates in THP-1-derived macrophages of each subtype (M0, M1, and M2, $n = 3$). *MALAT1* and pre-*GAPDH* were used as controls for nuclear RNA, and *ACTB* and *GAPDH* were used as controls for cytoplasmic RNA (see **Figure 2C**). (B) qRT-PCR measurement of vascular cell type-specific expression of lncRNA candidates ($n = 3$). THP-1_mono: Monocytic THP-1 cells, THP-1_M0: THP-1-derived macrophages, HCASMCs: Human coronary artery smooth muscle cells, HUVECs: Human umbilical vein endothelial cells. The RNA expression was normalized to *ACTB*. (C) The protein-coding potential of lncRNA candidates. The coding probabilities of lncRNA candidates were assessed by CPAT and CPC 2.0 tools. *MALAT1* was used as a control for the non-coding RNA, and *GAPDH* was used as a control for the protein-coding RNA (see **Figure 2D**). Data are presented as mean ± SEM. An unpaired two-tailed *t*-test with Welch's correction was used for statistical analysis. $^{**}p < 0.01$, $^{***}p < 0.005$, $^{****}p < 0.001$.

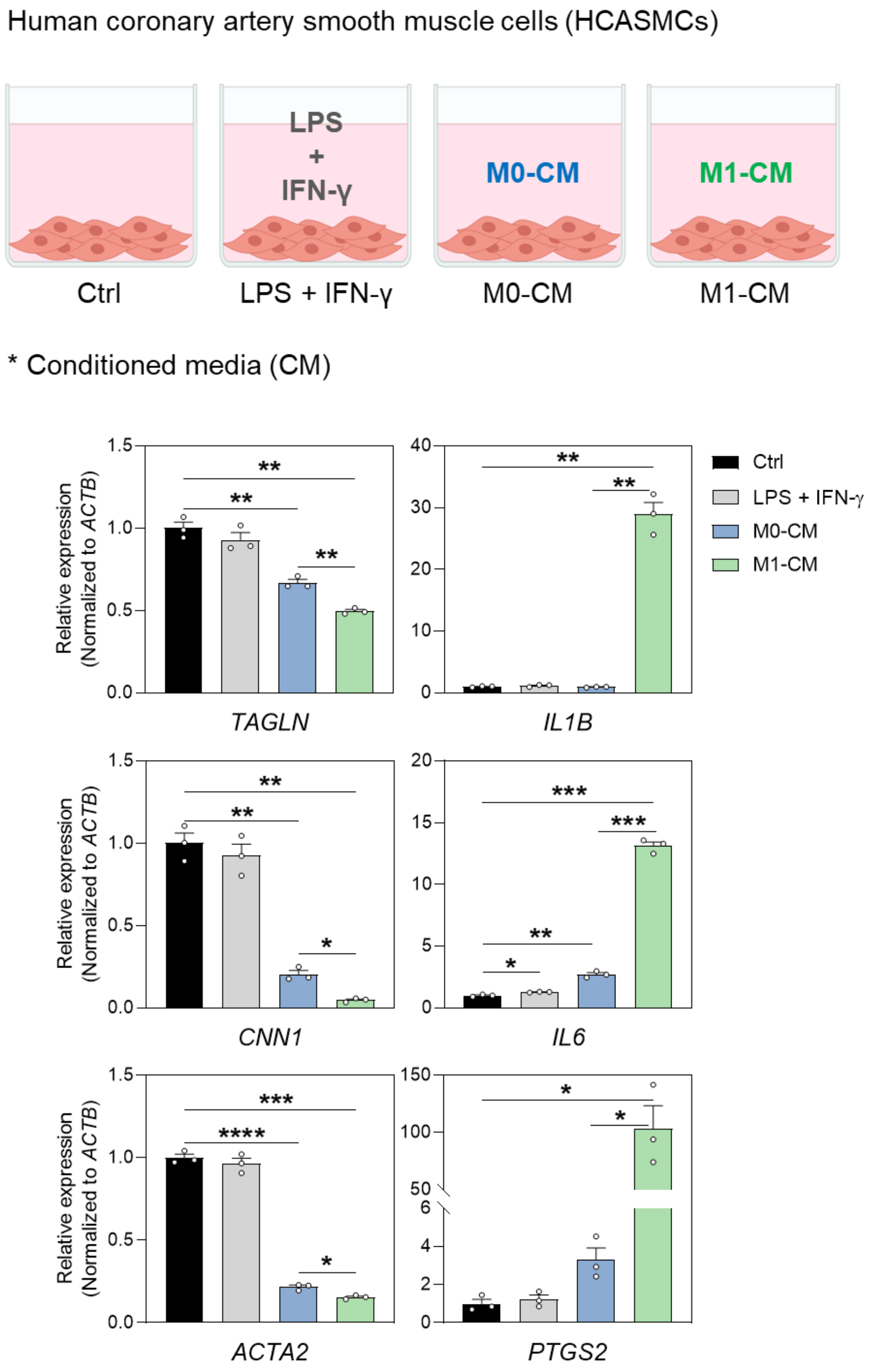

Figure S9. Establishing the treatment condition of conditioned media. Top: Each conditioned media (CM) was treated to HCASMCs for 24 hours. Ctrl (the media without treatment), LPS/IFN-γ (LPS and IFN-γ-added media incubated for one day without cells), M0-CM (the media cultured with M0 macrophages for one day), M1-CM (the media cultured with M1 macrophages for one day). Bottom: qRT-PCR measurement of contractile genes (*TAGLN*, *CNN1*, and *ACTA2*) and proinflammatory genes (*IL1B*, *IL6*, and *PTGS2*) after 24 hours of CM treatment ($n = 3$). The RNA expression was normalized to *ACTB*. Data are presented as mean ± SEM. An unpaired two-tailed *t*-test with Welch's correction was used for statistical analysis. *$p < 0.05$, **$p < 0.01$, ***$p < 0.005$, ****$p < 0.001$.

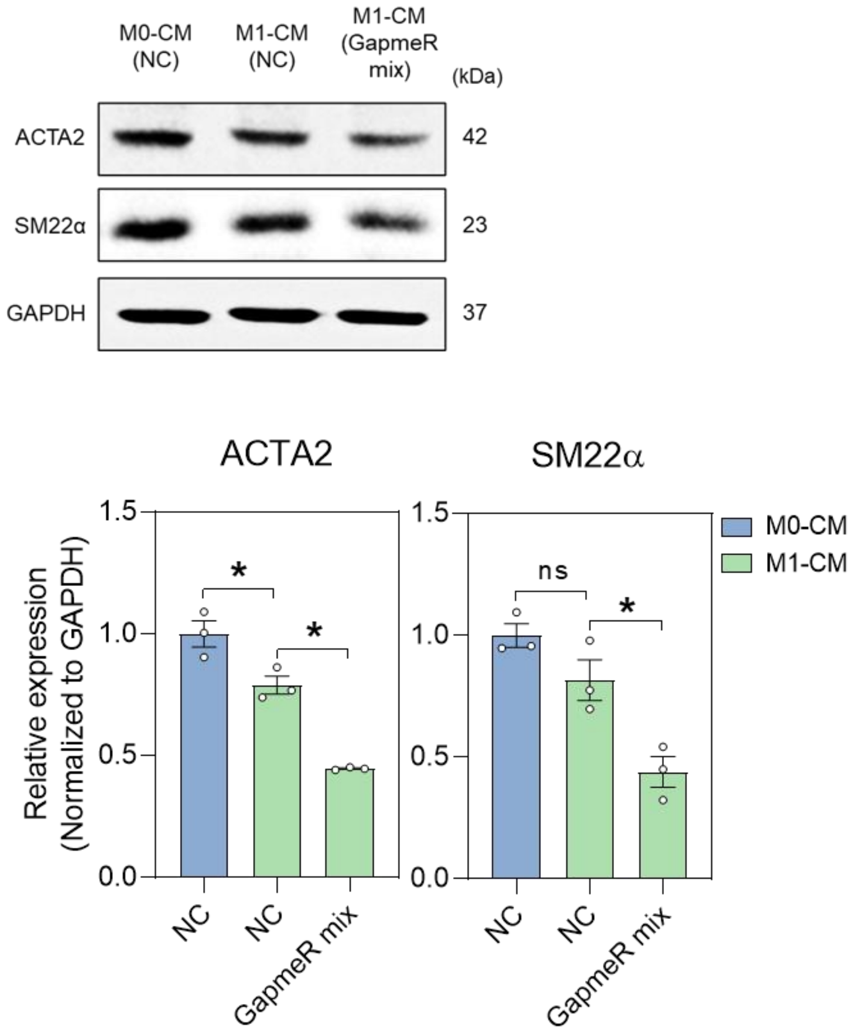

Figure S10. Effect of conditioned media of *SUGCT-AS1*-depleted THP-1 cells on the expression of contractile proteins in vascular smooth muscle cells. Western blot analysis of the expression of contractile proteins (ACTA2 and SM22*α*) in HCASMCs-treated with CM of THP-1-derived macrophages (M0 and M1) transfected with GapmeRs (NC and GapmeR mix) ($n = 3$). NC indicates negative control GapmeR. GapmeR mix is a mixture of GapmeR #1 and #2. The protein expression was normalized to GAPDH. Data are presented as mean ± SEM. An unpaired two-tailed *t*-test with Welch's correction was used for statistical analysis. *$p < 0.05$; ns: not significant.

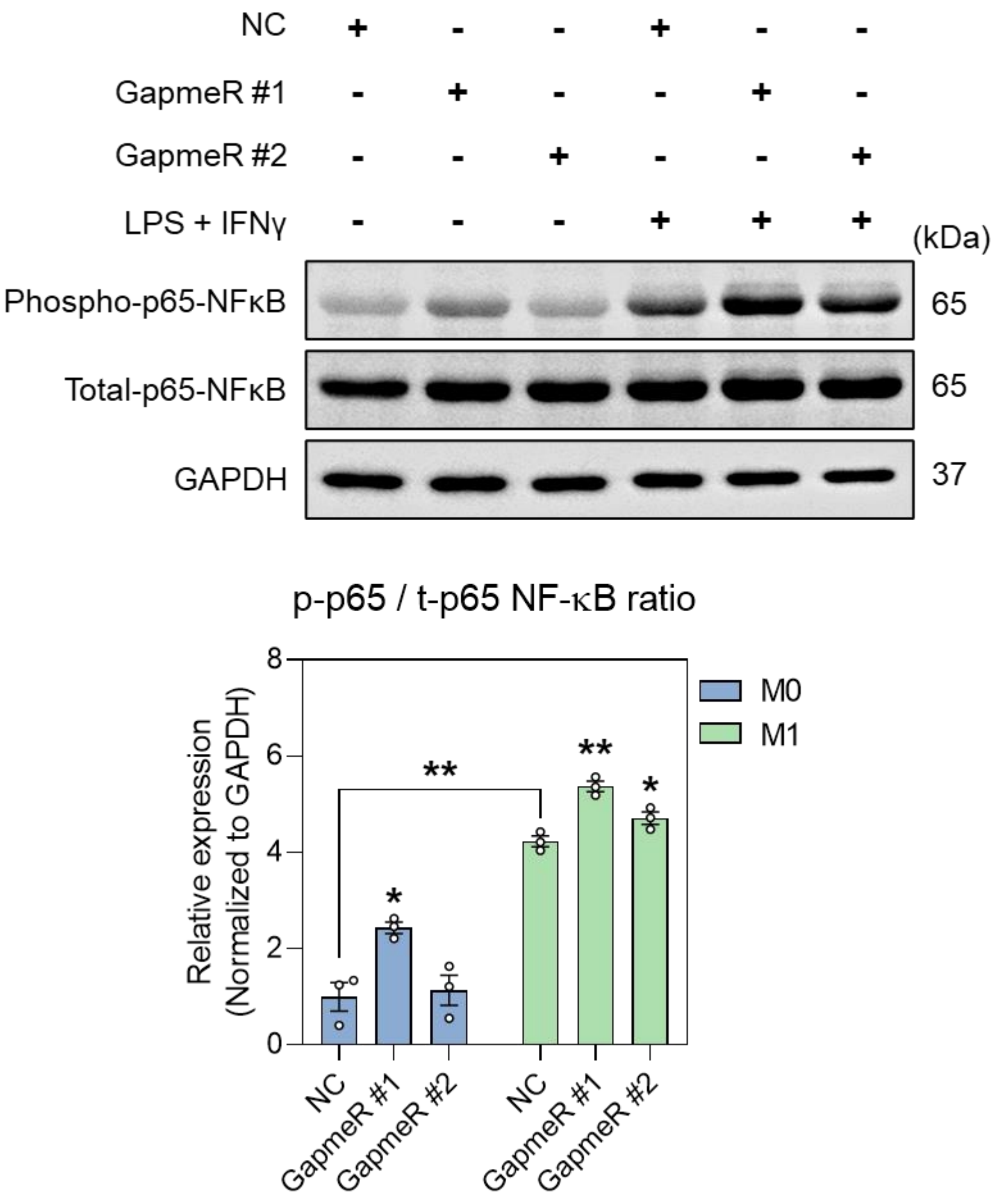

Figure S11. Effect of *SUGCT-AS1* knockdown on the activation of NF-κB signaling pathway. Western blot analysis of NF-κB p65 activation in THP-1-derived macrophages (M0 and M1) transfected with GapmeRs (NC, GapmeR #1, and #2) ($n = 3$). NC indicates negative control GapmeR. M1 stimulation was performed for 30 minutes. The ratio of phosphorylated p65 to total p65 was determined (phospho-p65/t-p65 ratio). The protein expression was normalized to GAPDH. Data are presented as mean ± SEM. An unpaired two-tailed *t*-test with Welch's correction was used for statistical analysis. *$p < 0.05$, **$p < 0.01$.

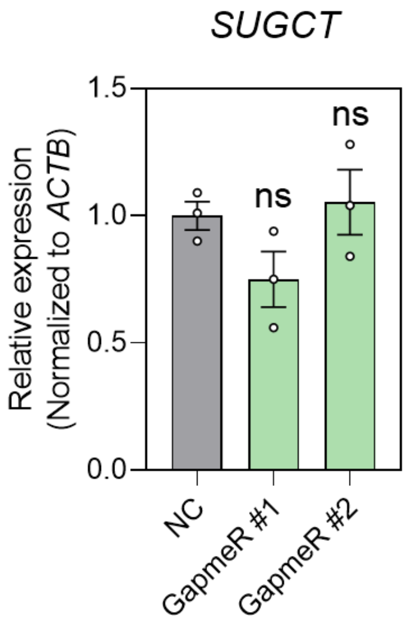

Figure S12. Effect of *SUGCT-AS1* knockdown on the expression of the neighboring gene. qRT-PCR measurement of *SUGCT* in *SUGCT-AS1*-depleted THP-1-derived M1 macrophages (n = 3). NC indicates negative control GapmeR. M1 stimulation was treated for 24 hours. The RNA expression was normalized to *ACTB*. Data are presented as mean ± SEM. An unpaired two-tailed t-test with Welch's correction was used for statistical analysis. ns: not significant.

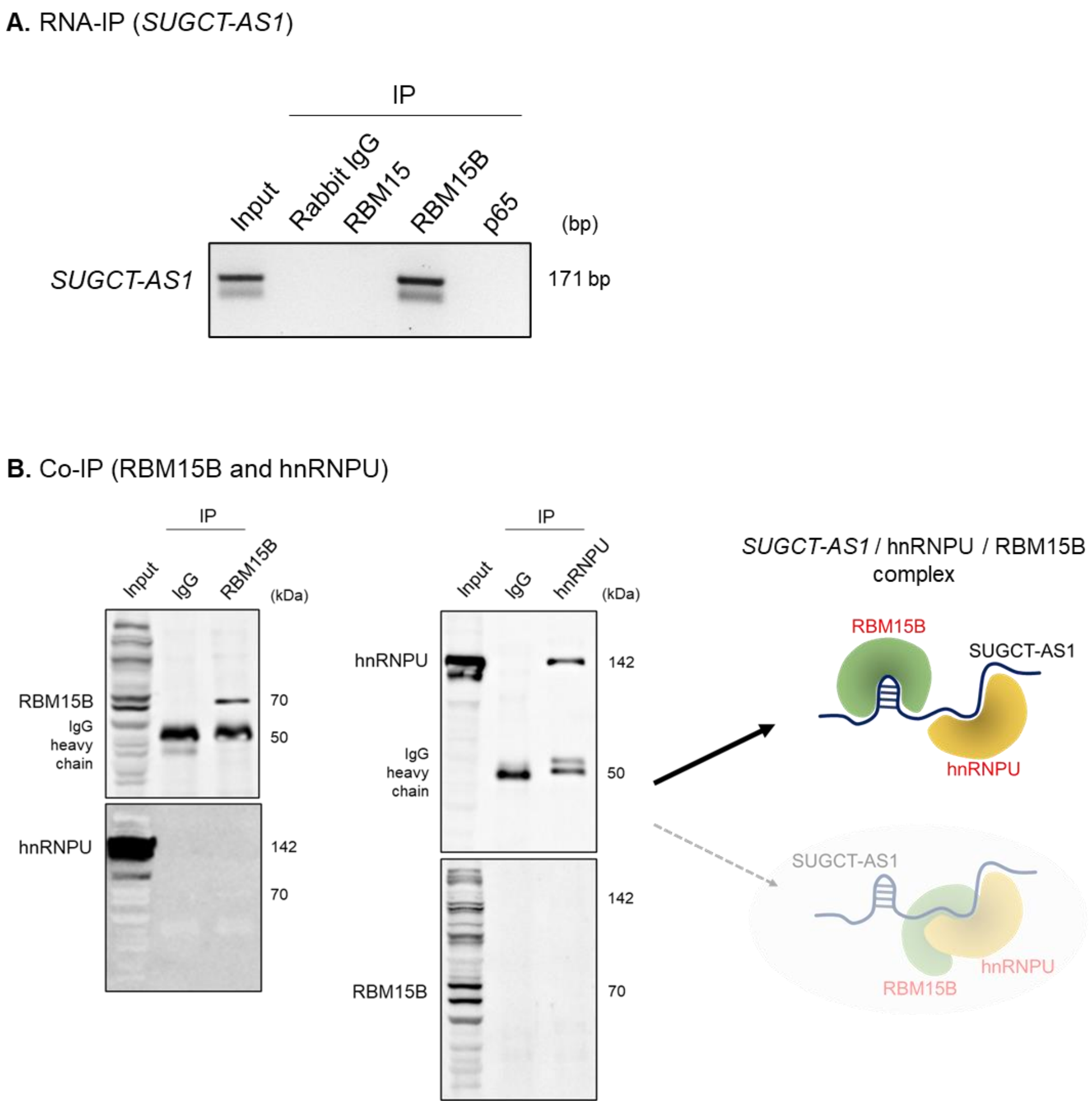

Figure S13. RNA-binding protein immunoprecipitation (RIP). (A) Confirmation of the interactions between *SUGCT-AS1* and predicted proteins (RBM15, RBM15B, and p65) in THP-1-derived M0 macrophages ($n = 3$). IP: Immunoprecipitation. (B) Co-immunoprecipitation (Co-IP) experiments to confirm whether each antibody used in this RIP efficiently binds to *SUGCT-AS1*-binding proteins (RBM15B and hnRNPU) and whether there is direct binding between RBM15B and hnRNPU. After blotting with each antibody (upper blot), the co-immunoprecipitation between RBM15B and hnRNPU was checked by attaching different antibodies (lower blot), after stripping the previous antibody.

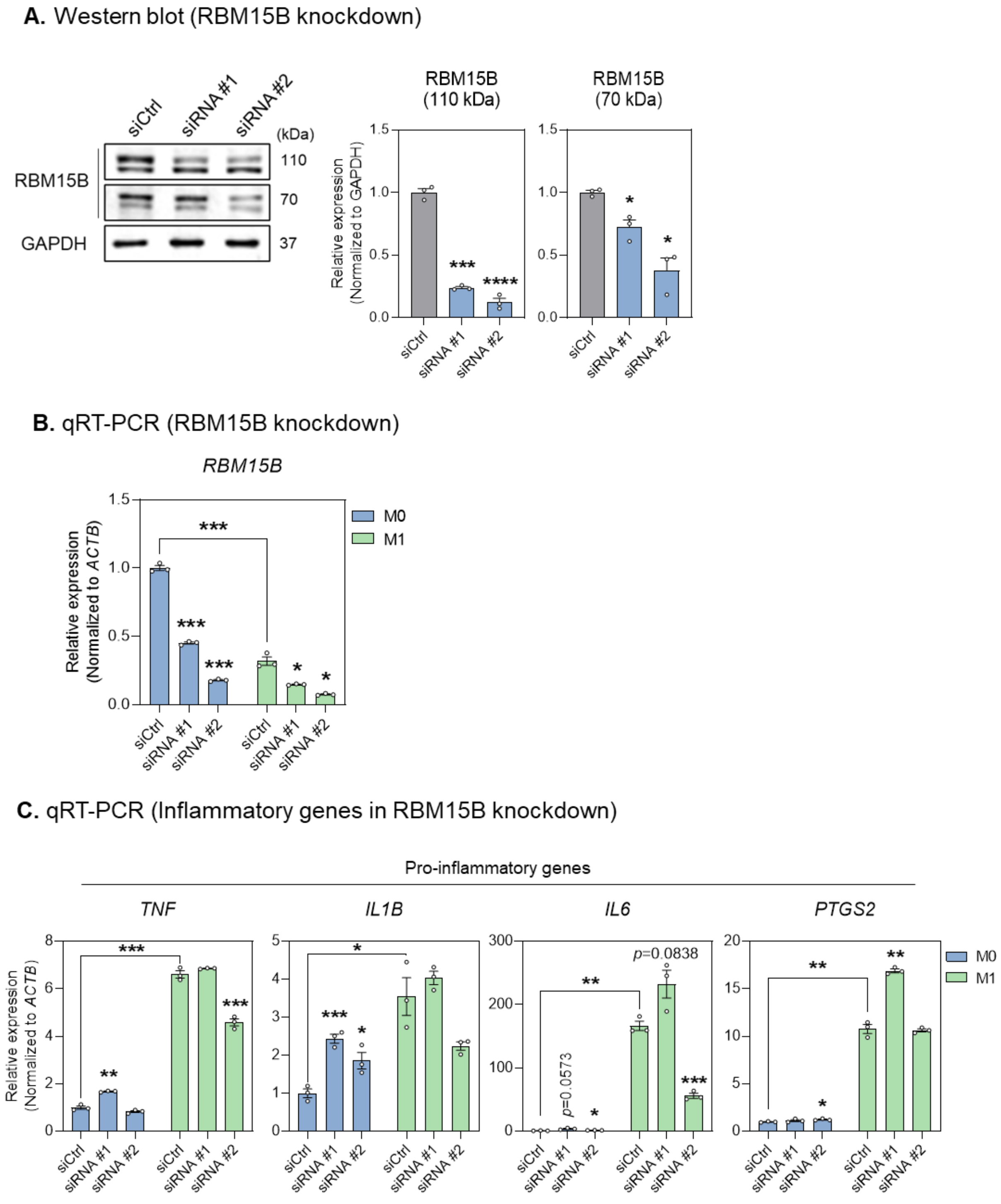

Figure S14. Effect of RBM15B knockdown on the expression of proinflammatory genes. (A) Verification of RBM15B knockdown by Western blot analysis in THP-1-derived M0 macrophages transfected with siRNAs (siCtrl, siRNA #1, and #2) for 24 hours (n = 3). The siRNAs against RBM15B effectively depleted both isoforms (110 and 70 kDa) of RBM15B. Bands marked with an asterisk are the target RBM15B. The siCtrl indicates the negative control siRNA. The protein expression was normalized to GAPDH. (B) Verification of *RBM15B* knockdown by qRT-PCR in THP-1-derived macrophages (M0 and M1) transfected with siRNAs for 24 hours. (C) qRT-PCR measurement of proinflammatory genes (*TNF*, *IL1B*, *IL6*, and *PTGS2*) in THP-1-derived macrophages (M0 and M1) transfected with siRNAs for 24 hours (n = 3). M1 stimulation was performed for 24 hours. The RNA expression was normalized to *ACTB*. Data are presented as mean ± SEM. An unpaired two-tailed t-test with Welch's correction was used for statistical analysis. *$p < 0.05$, **$p < 0.01$, ***$p < 0.005$, ****$p < 0.001$.

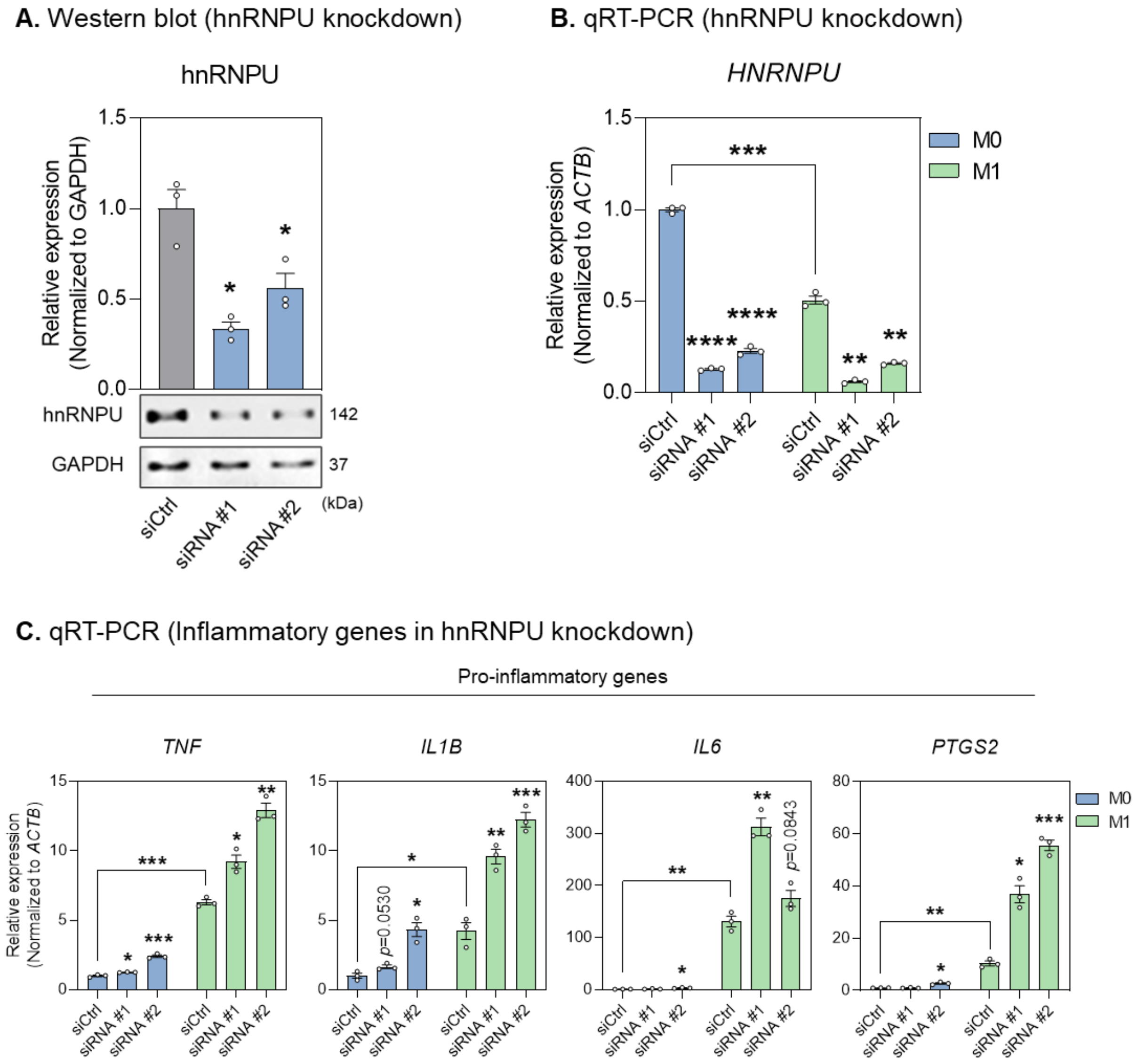

Figure S15. Effect of hnRNPU knockdown on the expression of proinflammatory genes. (A) Verification of hnRNPU knockdown by Western blot analysis in THP-1-derived M0 macrophages transfected with siRNAs (siCtrl, siRNA #1, and #2) for 24 hours (n = 3). The siCtrl indicates negative control siRNA. The protein expression was normalized to GAPDH. (B) Verification of *HNRNPU* knockdown by qRT-PCR in THP-1-derived macrophages (M0 and M1) transfected with siRNAs for 24 hours. (C) qRT-PCR measurement of proinflammatory genes (*TNF*, *IL1B*, *IL6*, and *PTGS2*) in THP-1-derived macrophages (M0 and M1) transfected with siRNAs for 24 hours (n = 3). M1 stimulation was performed for 24 hours. The RNA expression was normalized to *ACTB*. Data are presented as mean ± SEM. An unpaired two-tailed t-test with Welch's correction was used for statistical analysis. *$p < 0.05$, **$p < 0.01$, ***$p < 0.005$, ****$p < 0.001$.

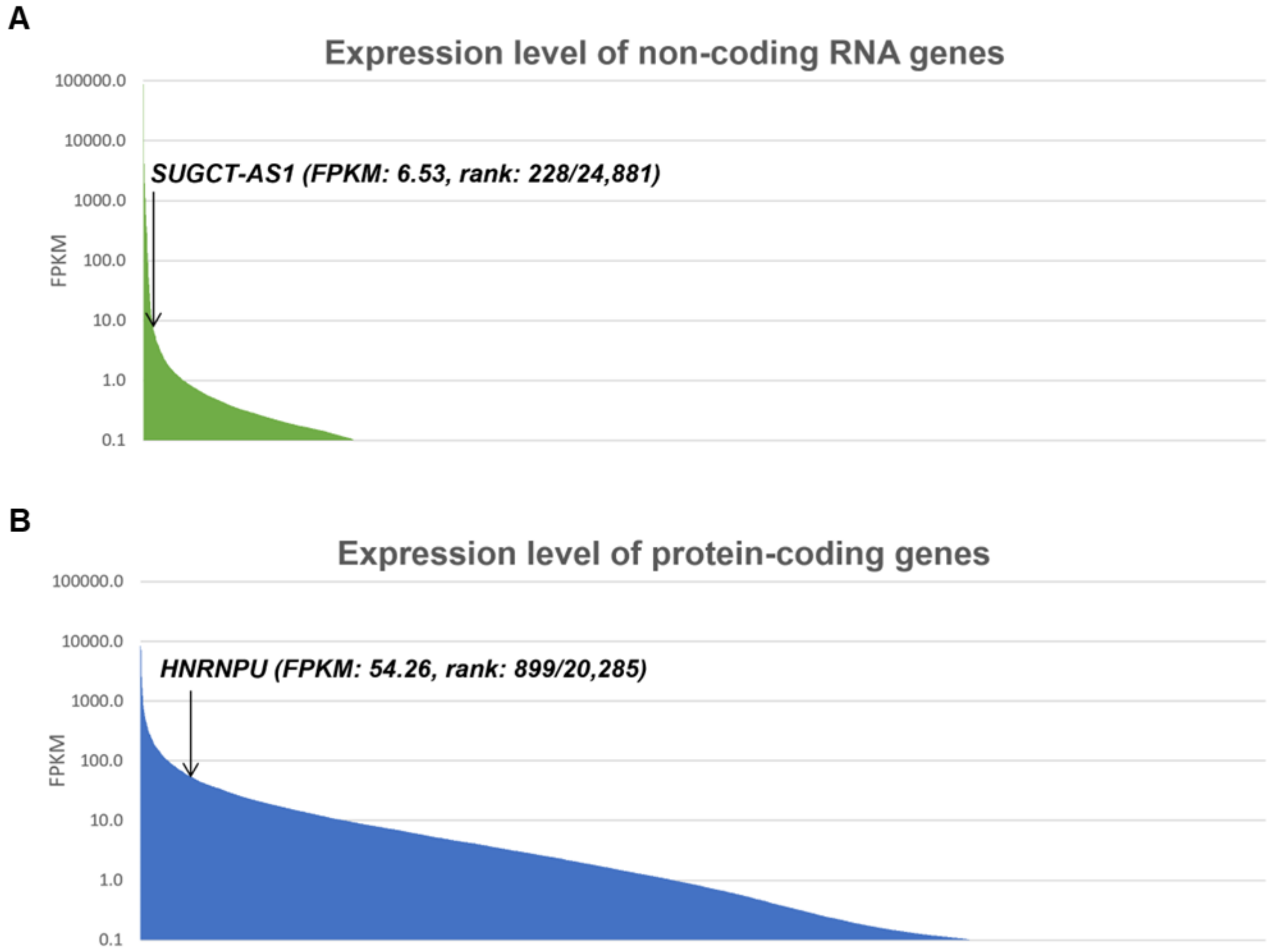

Figure S16. The relative expression level of *SUGCT-AS1* and *HNRNPU* in the THP-1-derived macrophages. The fragments per kilobase of the transcript per million mapped reads (FPKM) values of non-coding (A) and protein-coding genes (B) in THP-1-derived macrophages were depicted. According to the analysis, the expression of *SUGCT-AS1* ranked 228th out of 24,881 non-coding genes, and that of *HNRNPU* ranked 899th out of 20,285 protein-coding genes.

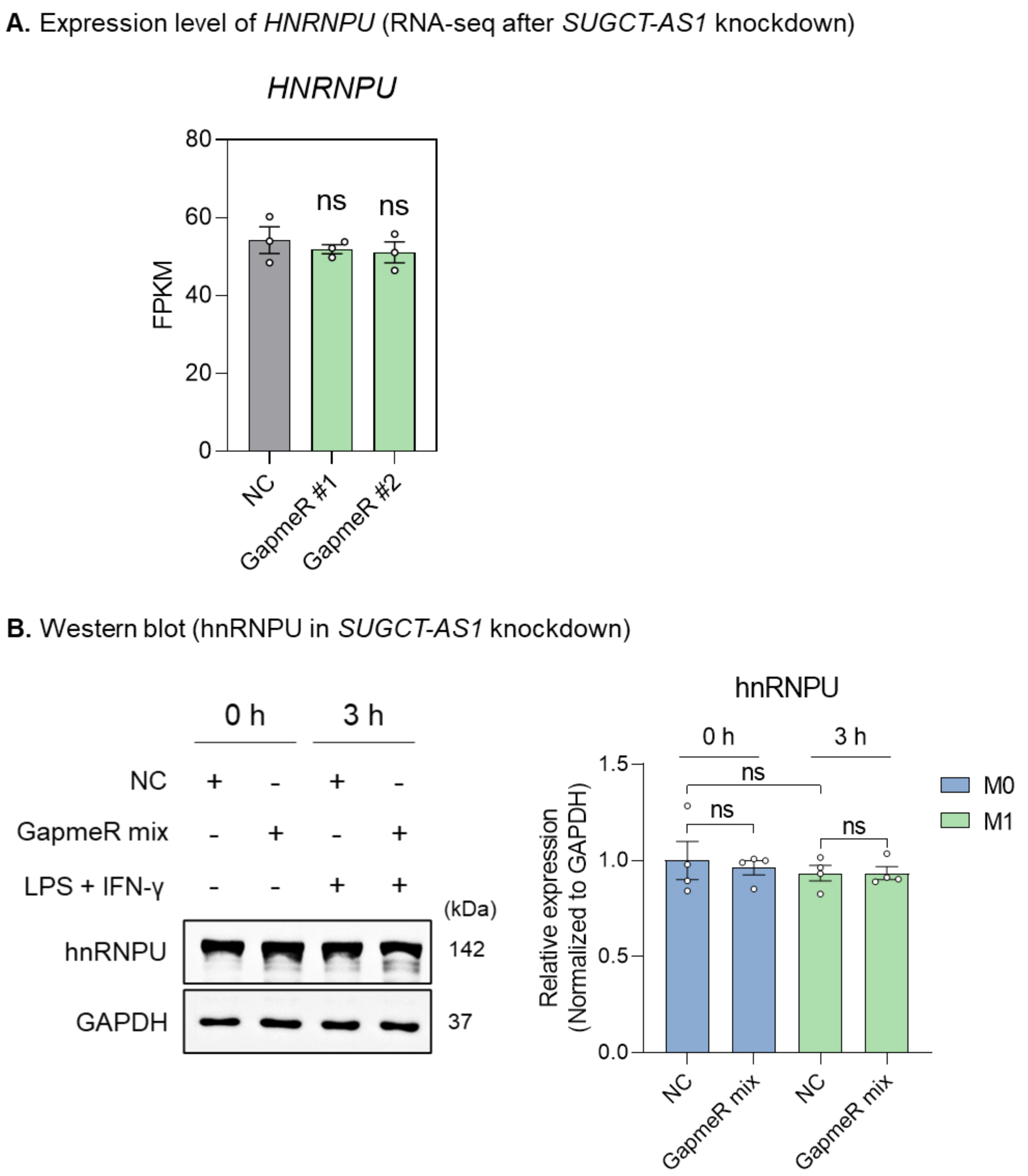

Figure S17. The expression of hnRNPU in *SUGCT-AS1*-depleted THP-1-derived macrophages. (A) FPKM values of *HNRNPU* in RNA sequencing data performed in *SUGCT-AS1*-depleted THP-1-derived M1 macrophages ($n = 3$). NC indicates negative control GapmeR. (B) Western blot analysis of the expression of hnRNPU in THP-1-derived macrophages (M0 and M1) transfected with GapmeRs (NC and GapmeR mix) ($n = 4$). M1 stimulation was performed for 3 hours. GapmeR mix is a mixture of GapmeR #1 and #2. The protein expression was normalized to GAPDH. Data are presented as mean ± SEM. An unpaired two-tailed *t*-test with Welch's correction was used for statistical analysis; ns: not significant.

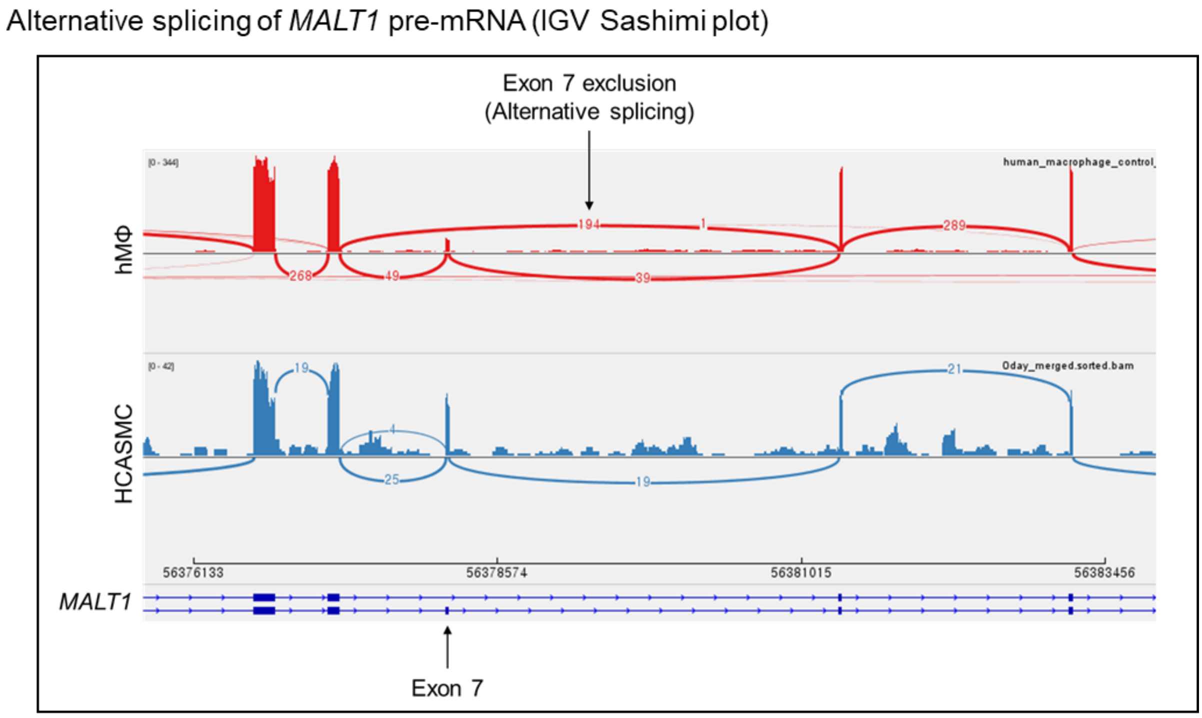

Figure S18. Alternative splicing of *MALT1* precursor mRNA. Alternative splicing of human *MALT1* precursor mRNA (pre-mRNA) was visualized using the Sashimi plot tool of the IGV genome browser (hg19) [43]. Our previous RNA-seq data for human coronary artery smooth muscle cells (HCASMCs) and public data for human CD14⁺ monocyte-derived macrophages (hMΦ, GSE101868) were utilized in this analysis [41, 42]. Arrows indicate the exclusion of MALT1's exon 7 in human macrophages.

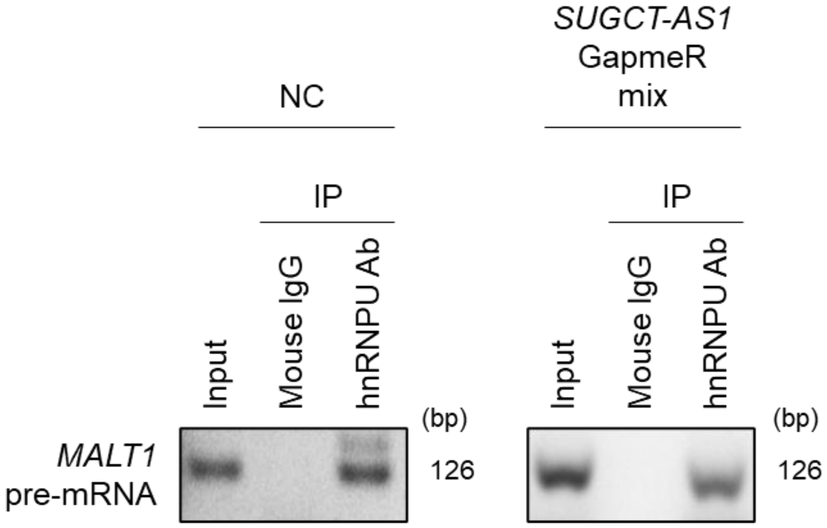

Figure S19. Depletion of *SUGCT-AS1* did not affect the binding of hnRNPU to *MALT1* precursor mRNA. RNA-binding protein immunoprecipitation (RIP) experiment of *MALT1* pre-mRNA using primary antibody against hnRNPU in THP-1-derived M0 macrophages transfected with GapmeRs (NC and GapmeR mix) ($n = 3$). NC indicates negative control GapmeR. GapmeR mix is a mixture of GapmeR #1 and #2 against *SUGCT-AS1*.

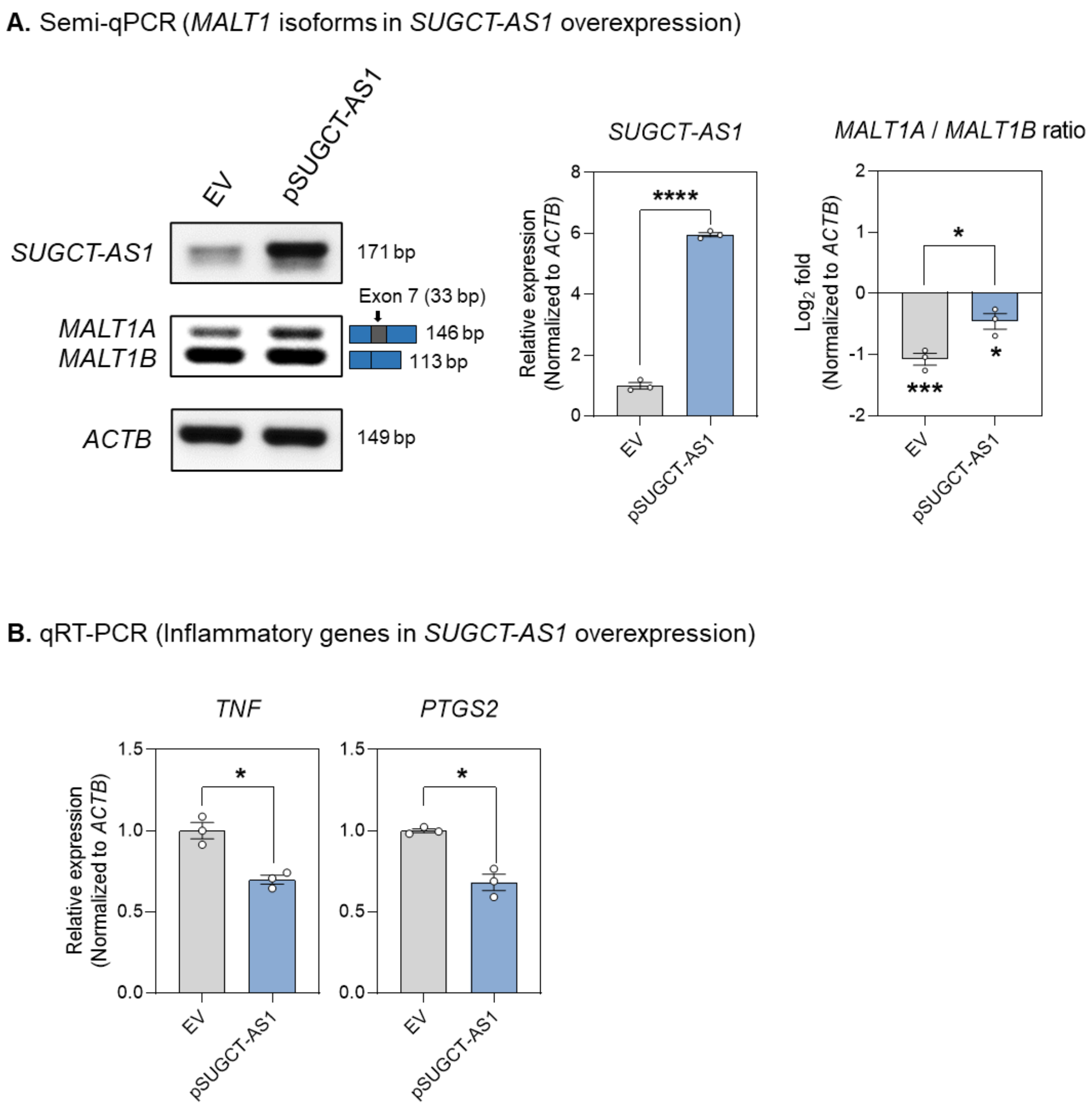

Figure S20. Effect of *SUGCT-AS1* overexpression in THP-1-derived macrophages. (A) Semi-qPCR measurement of *SUGCT-AS1* expression and the ratio of *MALT1* isoforms (*MALT1A* and *MALT1B*) in THP-1-derived M0 macrophages transfected with plasmid constructs (EV and pSUGCT-AS1) (n = 3). EV indicates an empty vector (pcDNA3). pSUGCT-AS1 is pcDNA3-*SUGCT-AS1*. *MALT1A*: Exon 7 included isoform; *MALT1B*: Exon 7 excluded isoform. (B) qRT-PCR measurement of proinflammatory genes in THP-1-derived M0 macrophages transfected with plasmid constructs (EV and pSUGCT-AS1) (n = 3). The RNA expression was normalized to *ACTB*. Data are presented as mean ± SEM. An unpaired two-tailed *t*-test with Welch's correction was used for statistical analysis. *$p < 0.05$, ***$p < 0.005$, ****$p < 0.001$.

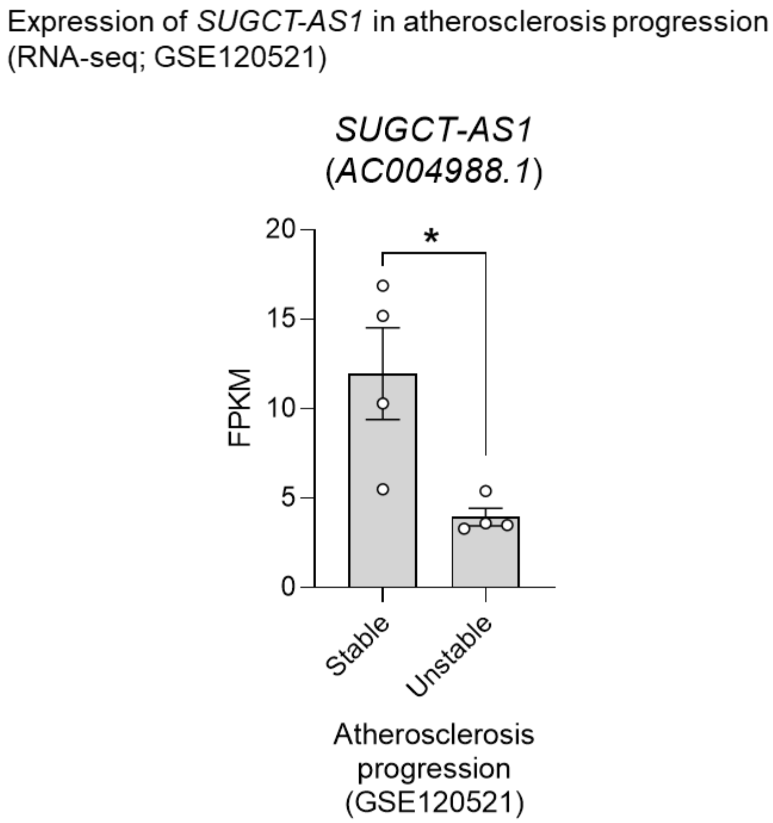

Figure S21. The expression of *SUGCT-AS1* in human atherosclerosis progression. The fragments per kilobase of the transcript per million mapped reads (FPKM) values of *SUGCT-AS1* (*AC004988.1*) in public RNA sequencing data for human atherosclerosis progression (GSE120521, $n = 4$) [19]. Stable plaques of atherosclerosis were used as a control and compared to unstable plaques. Data are presented as mean ± SEM. An unpaired two-tailed *t*-test with Welch's correction was used for statistical analysis. $^*p < 0.05$.
